# The oldest fossil bird-like footprints from the upper Triassic of southern Africa

**DOI:** 10.1371/journal.pone.0293021

**Published:** 2023-11-29

**Authors:** Miengah Abrahams, Emese M. Bordy

**Affiliations:** Department of Geological Sciences, University of Cape Town, Cape Town, South Africa; University of Silesia, POLAND

## Abstract

Footprint morphology reflects the anatomy of the trackmaker’s foot and is direct evidence for the animal’s behaviour. Consequently, fossil tracks can be used to infer ancient diversity, ethology, and evolutionary trends. This is particularly useful for deep-time intervals during which the early history of an animal group is reliant upon limited fossil skeletal material. Fossil tracks of early birds and theropods, the co-existing dinosaurian ancestors of birds, co-occur in the rock record since the Early Cretaceous. However, the evolutionary transition from dinosaur to bird and the timing of the birds’ origin are still contested. Skeletal remains of the basal-most birds *Aurornis*, *Anchiornis*, *Archaeopteryx* and *Xiaotingia* are Middle to Late Jurassic, while tracks with tentative bird affinities, attributed to dinosaurs, are known from as early as the Late Triassic. Here, we present numerous, well-provenanced, Late Triassic and Early Jurassic tridactyl tracks from southern Africa, with demonstrable bird-like affinities, predating basal bird body fossils by c. 60 million years.

## Introduction

Birds are one of the most diverse groups of animals on Earth with ~10 000 extant species [1], yet their early evolutionary history is still shrouded in mystery [2]. The dinosaurian origin of modern birds (Neornithes) unequivocally points to Maniraptora, a group of theropods [e.g., 3, 4], but the timing of the origin of birds is contested. The oldest body fossil record of basal birds comprises Middle to Late Jurassic (150–160 Ma) *Aurornis*, *Anchiornis*, *Archaeopteryx* and *Xiaontingia* [e.g., 5–8], while dinosaurian footprints with bird-like morphologies are known since the Late Triassic [e.g., 9–12]. The basal birds known from body fossils likely originated in the Early or pre-Jurassic, although, to-date, this is unconfirmed by the early osteological record that is exceedingly fragmentary. The only Late Triassic body fossil specimen posited to be bird-like is *Protoavis* [13], but this assessment is based on ambiguous material [3, 14] and is not widely accepted to be a basal bird. In this context, all ancient bird-like palaeontological discoveries are vital for unravelling both the origin of birds and the evolution of dinosaurs.

Multiple tridactyl tracks with a “proto-avian” morphology from the Upper Triassic to Lower Jurassic of southern Africa were assigned to the ichnogenus *Trisauropodiscus* in the mid-1900s [15–20] (S1 Table). Since its erection, the validity of the ichnogenus has been questioned [e.g.,^21^, ^22^], though recently there has been a resurgence of authors supporting Trisauropodiscus [12, 23], or at least the ichnospecies *T*. aviforma [24], as valid. Although recognizing *Trisauropodiscus* and its “proto-avian” affinity may potentially shift the origin of bird-like footprints by c. 60 million years, these tracks have essentially been disregarded for the last 50 years except for a handful of studies [e.g.,12, 23, 25, 26]. Based on the interpretive sketches and brief descriptions, the avian affinity of *Trisauropodiscus* has been hotly debated: some authors have likened the tracks to *Anomoepus* [e.g., 2, 21, 27, 28] accepted to be attributed to an ornithischian dinosaur, while others have agreed with its bird-like morphology [e.g., 22, 25], likening it to *Gruipeda* [22] which is attributed to plover-like birds.

**Fig 1:**
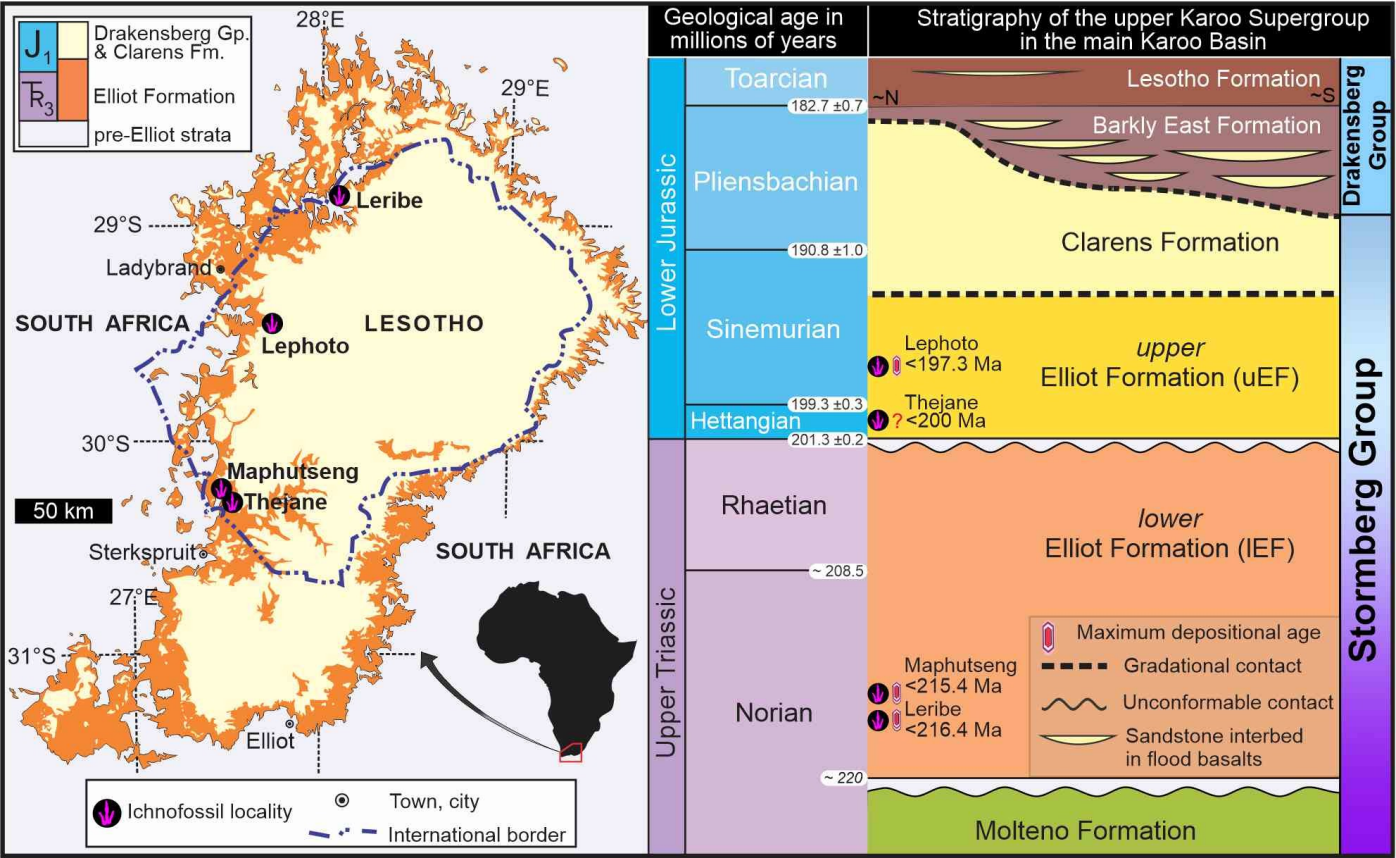
Geographic and stratigraphic location of southern African *Trisauropodiscus* ichnosites, modified after [29]. Detailed stratigraphic provenance of the ichnites is shown in S1 Table. Geological map has been modified from the Simplified Geology map (1:1000 000) of South Africa, Lesotho and Swaziland under a CC BY license, with permission from the CGS, original copyright [2003].

Here, we re-examine field material, original cast material, historical photographs and published interpretive sketches obtained from four locations in southern Africa (Fig 1) to reassess *Trisauropodiscus*. Our findings suggest that there are two distinct *Trisauropodiscus* morphotypes, one of which resembles footprints made by birds.

## Material and methods

**Fig 2:**
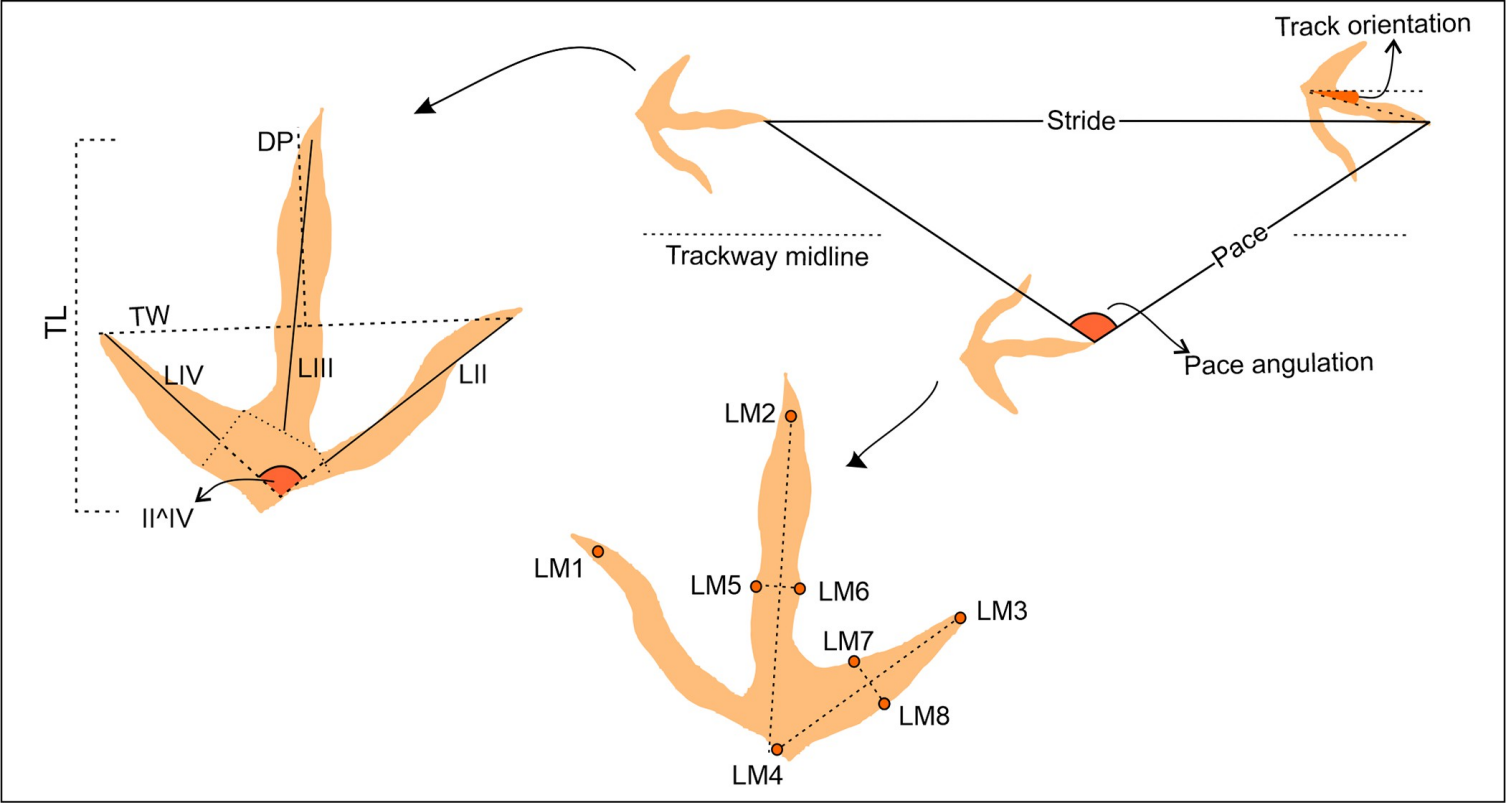
Measured parameters and landmark placement quantifying track morphology. Abbreviations: TL–track length, TW–track width, II^IV–total digit divarication, LM–landmark.

The track data considered in this study utilizes field material as well as cast material housed at the Université de Montpellier (France) and the Morija Museum and Archives (Lesotho), historical photographs and published interpretive sketches [16, 17, 18, 19] (see S1 Fig; S1–S3 Tables). The tracks are documented using standard, modern ichnological protocols [30] and no permits were required for the described study. Interpretative morphological outlines of the field and cast tracks were aided by false-colour depth maps produced using CloudCompare (version 2.6.1), which utilizes scaled 3D-photogrammetric models (for detailed methods, see [31]. Photogrammetric models and modelling data can be found in the Supplementary Materials (10.6084/m9.figshare.20291871). Standard tridactyl track and trackway parameters track length (TL), track width (TW), digit projection (DP), total digit divarication (II^IV), stride length, pace angulation (PA) and track rotation were measured on site or using ImageJ [32] (Fig 2; S2 and S3 Tables). To account for more of the track morphology, eight landmarks (LM) were identified and placed on morphologically well-defined field tracks and cast material (Fig 1). LM1 to LM3 were placed at the digit tip impressions (excluding claw marks) and LM4 at the proximal “heel” impression to account for TL, TW, LII-IV and II^IV. LM5-6 and LM7-8 were placed medially and perpendicularly to LM2 and LM4, and LM3 and LM4, respectively. This accounts for the digit width and allows the LMs to be placed consistently (digital pad impressions are lacking for most tracks and cannot be used as markers along the digit length). Landmarks were placed on perpendicular photographs using TpsUtil 1.75 and TpsDig software. A Procrustes fitting was applied to all the landmark data and the landmark-based principal component analysis was performed using PAST v3. The considered Ellenberger *Trisauropodiscus* morphotypes were compared to *Anomoepus* [33, 34] attributed to an ornithischian trackmaker; the *Grallator-Anchisauripus-Eubrontes* plexus [35], *Kayentapus* [36–38] and *Plesiornis* [9] attributed to theropod trackmakers; and *Aquatilavipes* [39, 40], *Avipeda* [41, 42], *Gruipeda* [22], *Jindongornipes* [42], *Quadridigitus* [42] attributed to avian trackmakers, as well as recently discovered *Trisauropodiscus* [12, 23]. A variance-covariance matrix was applied between groups with PC1 accounting 84% and PC2 accounting for 6% of the variation. Track orientation data was measured in ImageJ from original and historical field photographs, and analysed using Rose.Net, a freeware program for plotting directional rose diagrams and calculating vector statistics.

### Results

In the past two decades, we have revisited and redocumented numerous sites originally reported by Paul Ellenberger [e.g., 29, 31, 43–46] and from this are able to validate that his work from the mid-1900s is a fair representation of the relative stratigraphic placement and ichnological diversity. The original *Trisauropodiscus* were reported from four localities in Lesotho, and in our investigations, we successfully relocated the field material at Maphutseng (Fig 1), which we describe herein in more detail.

**Fig 3:**
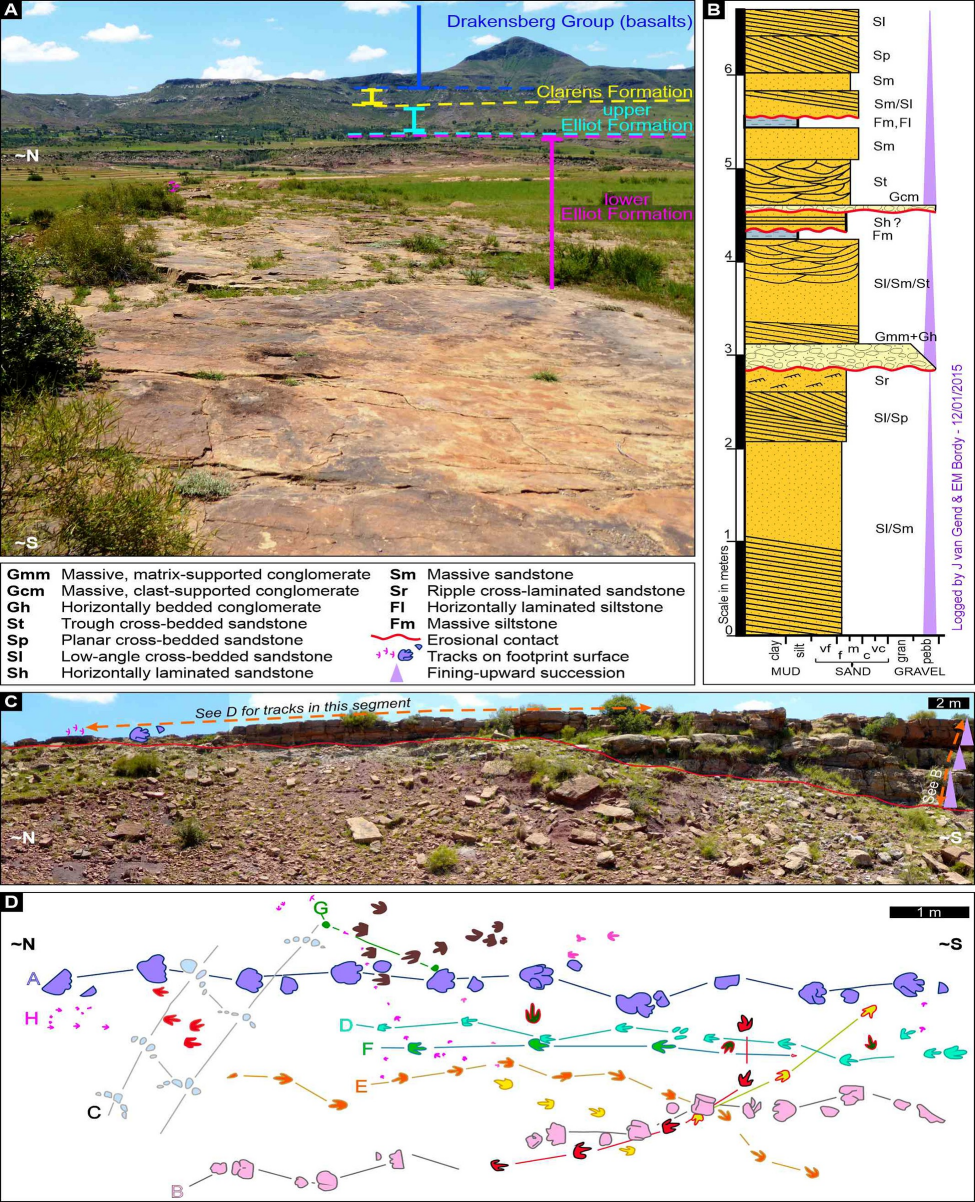
Sedimentological, stratigraphical and ichnological contexts of the Maphutseng tracksite. A) Local stratigraphy viewed from the footprint site, facing roughly north. B) Sedimentological log with facies codes of the sandstone-dominated, asymmetrical rock unit that immediately underlies the Maphutseng tracksite (see C for log’s position). C) N–S outcrop view of the stacked sandstone layers and multiple fining-upward successions (see log in B). Note the erosional contact of the sandstone unit and the underlying red, fossiliferous mudstone unit. See D for tracks documented by [47]. D) Maphutseng tracksite ichnodiversity showing at least 10 different individual footprint morphologies on a redrawn map of the original tracks from [47]. Note that cluster H and other magenta tracks mark *Trisauropodiscus* on the surface in C. The Maphutseng tracksite ichnodiversity map has been modified from [47] under a CC BY license, with permission from the Société Géologique de France, original copyright [1960].

The Maphutseng tracksite records a diverse range of ichnites (Fig 3), initially documented by [47], following its discovery by Paul Ellenberger in 1955. In our current and previous studies, we assessed the sedimentary architecture of the host rocks at the site and in the surrounding areas. Based on the sedimentological analysis and stratigraphic correlations in the region (Fig 3), the tracksite is located in the uppermost part of the lower Elliot Formation (lEF), approximately 20 meters below the lithostratigraphic contact with the overlying upper Elliot Formation (uEF). At Maphutseng, this lithostratigraphic boundary within the Elliot Formation is clearly defined by a mappable pedogenic nodule conglomerate unit, up to 2 meters thick, along with other sedimentary facies specific to the uEF. Compared to the uEF, the lEF is characterized by distinct lithofacies associations (Fig 3B), including differences in external and internal geometries of the sandstone units (Fig 3C), mineralogical composition, grain size, and palaeo-current patterns [48]. Although our litho- and biostratigraphic re-assessments suggest that the Maphutseng tracksite belongs to the uppermost part of the lEF, implying a Late Triassic age, a more precise age was also obtained by us through U-Pb LA-ICPMS age determinations of detrital zircons extracted from sandstones at the study site [29]. These results together with other maximum depositional ages obtained at tracksites across the outcrop area of the Elliot Formation in the main Karoo Basin are fully documented in [29] and references cited therein.

The Maphutseng footprints are preserved on the upper surface of an asymmetrical sandstone-dominated rock unit (Fig 3C). This unit consists mostly of laterally stacked sandstone layers with multiple fining-upward successions (Fig 3B), which is typical of the laterally accreted, meandering channel deposits common in the lEF [29, 48]. Along a major erosional contact, the sandstone unit is incised into the underlying red mudstone unit (Fig 3C). The same mudstones, which have yielded several hundred fossil bones, are known for having hosted the famous Maphutseng dinosaur cemetery [49], which is one of the laterally most extensive fossil-rich layers ever discovered, and now fully excavated, in upper Triassic of southern Africa.

The track-bearing palaeosurface spans ~80 meters in length and ~5 meters in width, and its extensive documentation was provided by [17]; however, neither the Ellenbergers’ descriptions, including published and unpublished photographs, nor our field investigations revealed any evidence of ancient microbial surfaces or water-saturated substrates in the northern extreme of the surface, where *Trisauropodiscus* tracks are most common (Fig 3A–3D). Desiccation cracks, on the other hand, are commonly found (S1 Fig) and are preserved both among the tracks and propagating from them in this sector of the palaeosurface. In the latter case, the relative timing of track and crack formation is unequivocal, with the tracks predating the cracks [50]. In the southern sector of the track-bearing palaeosurface, evidence of ancient microbial surfaces is lacking, however water-saturated substrates were likely more common, as indicated by sediment collapse features associated with tracks. The decrease in water saturation levels from south to north aligns with the overall sedimentological evidence at the site, suggesting that the palaeosurface likely represents the surface of a meander point bar that was more emergent in the north relative to the south (Fig 3).

**Fig 4:**
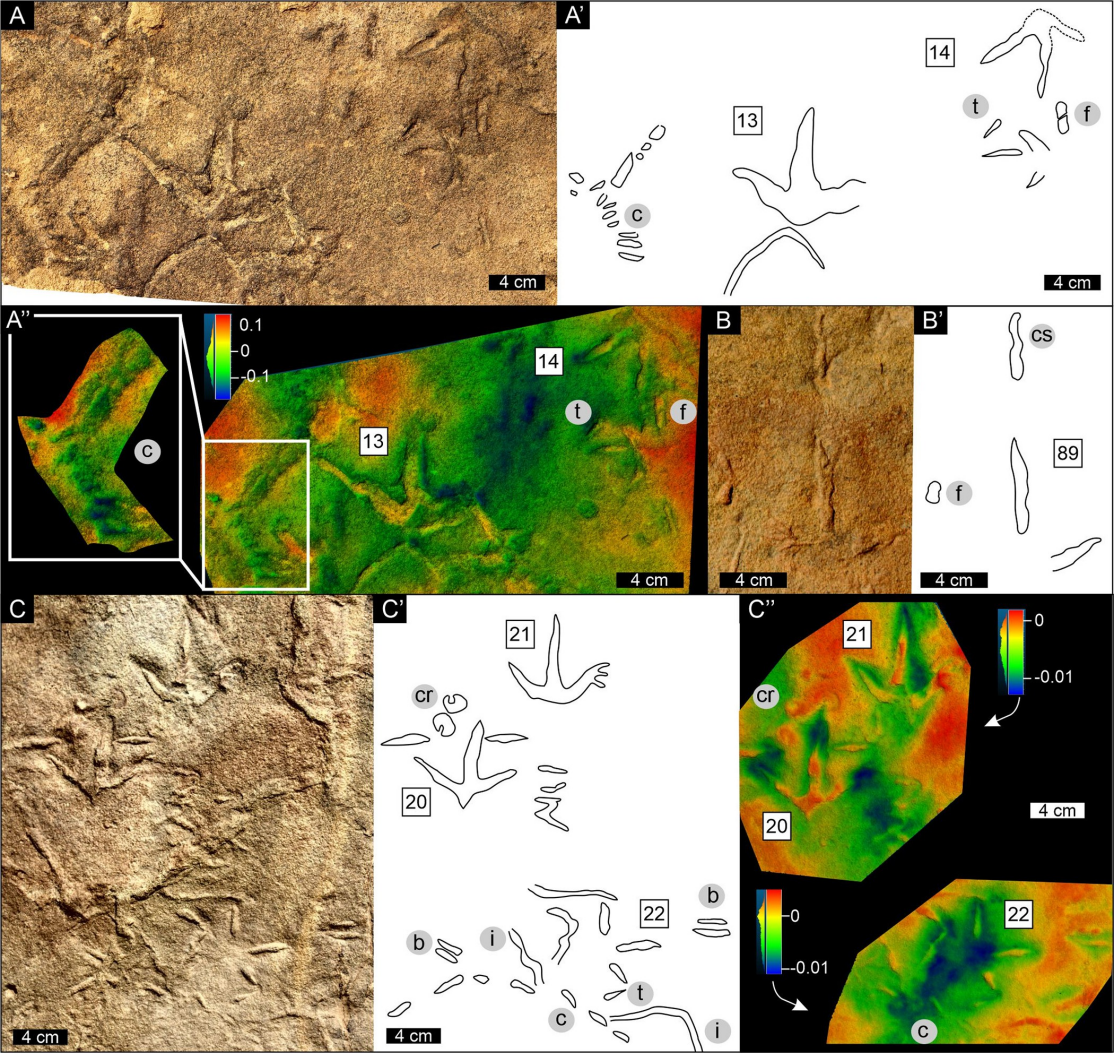
Photographs, interpretive outlines and false-colour depth maps of distinct traces associated with *Trisauropodiscus* at Maphutseng. False colour depth maps are applied to 3D photogrammetric models, where blue and red reflect negative and positive relief, respectively, relative to the average relief. See Methods for details. A, A’, A”) Markings resembling either pecking and probing activity, partial track (digit) impressions or invertebrate traces. B, B’, B”) Markings resembling either a continuous pecking trace or invertebrate trace. C, C’, C”) Unequivocal invertebrate trace and markings resembling either pecking and probing activities, or partial track (digit) impressions or invertebrate traces. Abbreviations: b–bilobate trace, c–chain-like trace, cr–crescent ring-like trace, cs–curviform-to-sinuous trace, f–figure of eight-like trace, i–invertebrate trace, t–teardrop-like trace.

Six discrete, herein unidentified, trace-like markings are exclusively preserved on the northern-most surface in association with *Trisauropodiscus* on the Maphusteng track-bearing palaeosurface (Fig 4). These markings, preserved as casts, have teardrop-like [51–53], figure of eight-like [51, 52], crescent-to-ring-like [54], curviform-to-sinuous [54], chain-like [42, 54], and bilobate morphologies. Although certain markings may appear similar to feeding traces resulting from bird pecking and probing, their interpretation remains unresolved. This is due not only to their resemblance to invertebrate traces but also because some, such as the teardrop and sinuous shapes, may be partial, overprinted, weathered or eroded track impressions.

**Fig 5:**
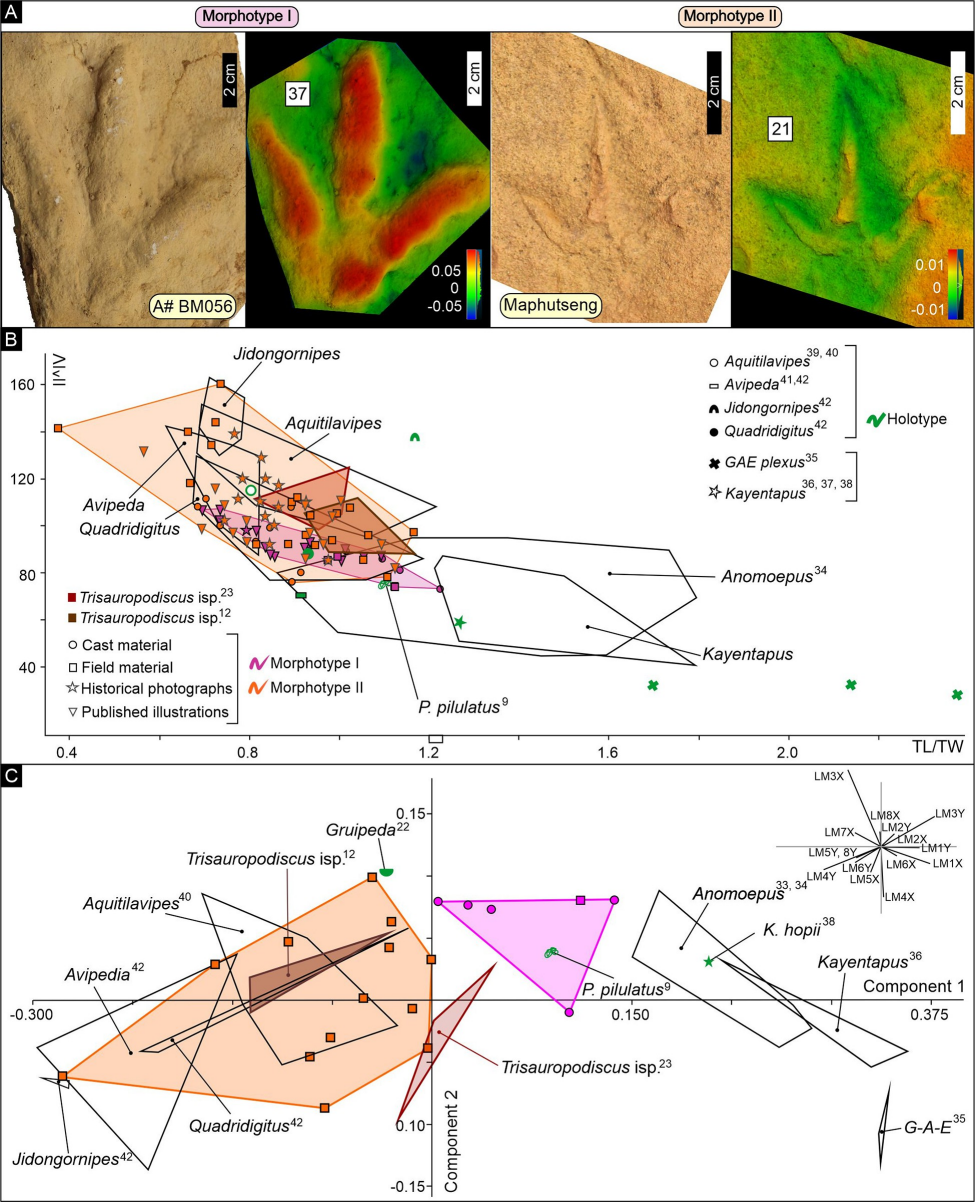
Morphological comparison of southern African *Trisauropodiscus* morphotypes with established ichnogenera attributed to avian, ornithischian and theropod trackmakers. Morphotypes I and II are largely indistinguishable when considering their track length-to-width ratio and total digit divarication, though Morphotype I generally has lower total digit divarication angles. When considering additional features using landmark-based principal component analysis, the two morphotypes form distinct fields. Although Morphotype I resembles *Anomoepus*, when plotted, the two ichnogenera fields do not overlap. Morphotype II morphospace consistently corresponds to established avian ichnogenera. Notably, both morphotypes are discrete from the classical, contemporaneous theropod ichnogenera *Kayentapus*, *Grallator*, *Anchisauripus* and *Eubrontes*. A) Photograph and false colour depth map of a typical Morphotype I and Morphotype II track. Key differences include larger track size, more robust digits and smaller total digit divarication of Morphotype I relative to Morphotype II. False-colour depth maps are applied to 3D photogrammetric models, where blue reflects negative relief and red reflects positive relief relative to the average relief. See Methods for details. B) Length-to-width ratio and total digit divarication angle plot. Measurements for the southern African morphotypes were performed by a single operator on > 80 tracks comprising field material (27 tracks), cast material (13 tracks), historical photographs (15 tracks), and published sketches (34 tracks, Table 1; S1 and S2 Figs, S1 Table). See Methods for details and S2 Fig for the track numbers corresponding to the data points. C) Landmark-based principal component analysis. Eight landmarks were placed on orthophotographs of the *Trisauropodiscus* morphotypes and comparative ichnotaxa by a single operator to account for additional morphological features such as digit thickness and anterior projection of the central digit. The morphospaces are most strongly controlled by landmarks, which are influenced by track width, total digit divarication and digit thickness. See Methods for details and S2 Fig for the track numbers corresponding to the data points. Abbreviations: TL–Track length, TW–Track width, II-IV–Digit numbers, II^IV–digit divarication angle.

Seven ichnospecies, based on varying morphological features, were originally assigned to the *Trisauropodiscus* ichnogenus [15–20]. Based on size, track length-to-width ratios, and digit morphologies of the holotype material, we assign them to two distinct morphotypes (Fig 5; S1 Fig; S1 and S2 Tables): Morphotype I, comprising *T*. *galliforma*, *T*. *levis*, *T*. *popompoi*, and *T*. *superaviforma*, is marginally longer than wide with robust digits that have a smaller total digit divarication, while Morphotype II, comprising *T*. *aviforma*, *T*. *phasianiforma*, and *T*. *superavipes*, is wider than long with slender digits and a larger total digit divarication.

**Table 1:** Track measurements for field material, cast material, historical photographs and illustrated sketches of *Trisauropodiscus* morphotypes I and II. See S1 Fig and S1 and S2 Tables for details.

		TL	TW	TL/TW	DP	DP/TW	II^IV			TL	TW	TL/TW	DP	DP/TW	II^IV
**Field material**								**Historical photographs**							
	Min	4.30	3.82	1.00	1.55	0.41	74								
Morphotype I	Max	4.55	4.28	1.13	2.02	0.47	87	Morphotype I							
(n = 3)	Mean	4.38	4.05	1.07	1.79	0.44	81	(n = 0)							
	Std. dev	0.14	0.33	0.09	0.33	0.05	9								
	Min	1.47	3.64	0.38	0.62	0.16	78		Min	1.97	2.54	0.75	1.07	0.27	85
Morphotype II	Max	5.19	7.69	1.17	4.17	0.96	160	Morphotype II	Max	11.74	14.60	0.98	3.93	0.65	139
(n = 23)	Mean	3.90	4.46	0.88	2.15	0.49	109	(n = 14)	Mean	3.85	4.64	0.84	2.16	0.50	110
	Std. dev	0.94	0.93	0.18	0.63	0.16	22		Std. dev	2.31	2.91	0.06	0.81	0.10	14
**Cast material**								**Interpretive sketches**							
	Min	3.75	3.28	0.94	1.56	0.33	73		Min	1.50	1.75	0.70	0.18	0.10	85
Morphotype I	Max	11.70	12.50	1.23	4.48	0.48	92	Morphotype I	Max	24.81	26.54	1.10	10.60	0.51	107
(n = 6)	Mean	9.11	8.16	1.09	3.33	0.43	84	(n = 18)	Mean	6.11	6.70	0.89	2.77	0.39	93
	Std. dev	3.30	3.61	0.11	1.15	0.06	7		Std. dev	5.89	6.07	0.11	2.66	0.11	7
	Min	2.27	2.76	0.69	1.14	0.37	76		Min	1.98	1.75	0.57	0.73	0.35	82
Morphotype II	Max	7.23	8.03	0.92	3.29	0.44	111	Morphotype II	Max	12.25	16.41	1.13	5.95	0.57	132
(n = 7)	Mean	3.59	4.31	0.83	1.75	0.41	96	(n = 16)	Mean	3.83	4.51	0.88	1.95	0.45	101
	Std. dev	1.66	1.75	0.09	0.71	0.02	14		Std. dev	2.37	3.26	0.15	1.13	0.07	13
**Field and cast material**								**Photographs and sketches**							
	Min	3.75	3.28	0.94	1.55	0.33	73		Min	1.50	1.75	0.70	0.18	0.10	85
Morphotype I	Max	11.70	12.50	1.23	4.48	0.48	92	Morphotype I	Max	24.81	26.54	1.10	10.60	0.51	107
(n = 9)	Mean	7.54	6.99	1.08	2.89	0.43	83	(n = 19)	Mean	6.11	6.70	0.89	2.77	0.39	93
	Std. dev	3.52	3.57	0.10	1.21	0.05	7		Std. dev	5.89	6.07	0.11	2.66	0.11	7
	Min	1.47	2.76	0.38	0.62	0.16	76		Min	1.97	1.75	0.57	0.73	0.27	82
Morphotype II	Max	7.23	8.03	1.17	4.17	0.96	160	Morphotype II	Max	12.25	16.41	1.13	5.95	0.65	139
(n = 30)	Mean	3.83	4.42	0.87	2.05	0.47	106	(n = 30)	Mean	3.84	4.57	0.86	2.05	0.47	105
	Std. dev	1.12	1.14	0.16	0.66	0.14	21		Std. dev	2.30	3.04	0.12	0.98	0.09	14

On average, Morphotype I tracks are larger than Morphotype II (6.59±5.19 cm vs 3.83±1.80 cm) but their distinct morphological characteristics are unlikely attributed to allometric changes as morphologies typifying Morphotype II are also preserved in larger tracks with track lengths typical of Morphotype I and vice versa (e.g., tracks 10 and 39; S1 Fig; S2 Table). Both morphotypes are known from both Late Triassic and Early Jurassic ichnosites and commonly co-occur on these track-bearing surfaces (Figs 3 and 4; S1 Fig; S1 Table). While the morphological differences between Morphotype I and II could justify their classification into separate ichnogenera, this study does not include any ichnotaxonomic assessment or revision of the diagnosis of *Trisauropodiscus*.

A total of 163 *Trisauropodiscus* tracks from field and historical photographs were evaluated on 11 individual track-bearing slabs from the Maphutseng and Thejane track sites to determine the track orientation and density (see Methods; S4 and S5 Tables). The average track number per slab is 10, when disregarding the lowest and highest track numbers, which are 4 and 64, respectively. On each slab, the tracks are directed at random, and this lack of orientation is well illustrated by the vector statistics. Track directional data has a standard deviation range of ~ 18°–80° and a consistency ratio of 0.01–0.95. When disregarding the lowest and highest values, the average standard deviation and consistency ratio of the directional data is ~ 66° and ~ 0.49, respectively. The vector statistics of the slab with the highest track number (n = 64) shows the highest standard deviation and lowest consistency ratio at 80.42° and 0.01, respectively.

### Morphotype I

Morphotype I tracks are documented from the Maphutseng field ichnosite, cast material, and interpretive sketches (Figs 1 and 3; Table 1; S1 Fig; S1–S3 Tables). At Maphutseng, the isolated, asymmetrical tracks are preserved as slightly longer than wide (mean TL/TW of 1.07±0.09) shallow hyporeliefs. The digit impressions (LII<LIII<LIV) are relatively robust, with rounded tips and pad impressions preserved in tracks 9 and 10, and claw marks preserved in track 10 (S1 Fig). Where digital pads are present, the proximal digit IV pad is distinctly aligned with the central axis of digit III. Cast materials preserve the same morphology, with a digital pad formula of 2-3-4 clear in track 37 (repository A#BM056; Fig 5). Comparable moderate average total digit divarication angles are preserved for the field and cast material (84° vs 81°; Table 1; S2 Table). A single, bipedal Morphotype I trackway is documented in repository A#G041 (tracks 33 to 35; S1 Fig) with the tracks having a slight rotation relative to the midline (track 33–6°, track 34 4°), a large pace angulation of 151°, and a stride 6 times the track length (S3 Table). Interpretive sketches of Morphotype I have measured parameters within a single standard of deviation overlap with the measured parameters of the field and cast material, but on average, the illustrated material is wider and has larger digit splays (Fig 5; Table 1; S1 Fig; S1 Table). The sketched trackways are also bipedal, with average track rotations of -13°, pace angulations of 174° and stride 8 times the track length (S3 Table).

The wide and round digit morphology of Morphotype I, with the digit IV proximal pad aligned with digit III, resembles *Anomoepus* [33, 34], a contemporaneous tridactyl track. The pes impression of *Anomoepus* is small (track length < 20 cm), asymmetrical, and distinctly preserves digit IV’s metatarsophalangeal pad in line with digit III. *Anomoepus* digit impressions are cigar-shaped with LII < LIII < LIV and the outer digits have moderate total digit divarication (~ 68°). Furthermore, Morphotype I trackways preserve pace angulations and inward track rotations consistent with *Anomoepus* [55].

When considering measured parameters (TL/TW vs II^IV), Morphotype I is indistinguishable from Morphotype II, but generally preserves lower total digit divarication, which is most clearly seen when considering field and cast material (Fig 5). Morphotype I field and cast material TL/TW and II^IV coincides with *Anomoepu*s, while the historical photographs and published illustrations depict forms that are wider with more splayed digits than *Anomoepus*. When considering landmark-based principal component analysis which accounts for more of the track morphology, Morphotype I predominantly plots in the same quadrant as *Anomoepus* and *Kayentapus* (Fig 5). This quadrant is most strongly influenced by landmarks 1, 3 and 4, directly related to the digit II and IV ends i.e., track width, “heel” and total digit divarication. Although in the same quadrant, Morphotype I morphospace does not coincide with *Anomoepus* or *Kayentapus* i.e., Morphotype I is distinct from *Anomoepus* and *Kayentapus*. The holotype *P*. pilulatus [9] plots within the Morphotype I morphospace; however, Morphotype I never preserves hallux impressions nor a pronounced digit III metatarsophalangeal pad and does preserve defined digital pad impressions which is dissimilar from *P*. *pilulatus*.

Based on Ellenberger’s works, some authors consider *Trisauropodiscus* to be a junior synonym of *Anomoepus* [e.g., 21], while others [25], several of whom have recently documented new field specimens [e.g., 12, 23, 26], have argued *Trisauropodiscu*s is a distinct ichnotaxa. It is worth noting that in Ellenberger’s original report on the Maphutseng tracksite [47], he noted that the small tridactyl tracks resembled *Anomoepus* but still considered them as distinct. Our principal component analysis supports Ellenberger and these more recent authors who posit *Trisauropodiscus* is distinct from *Anomoepus*.

### Morphotype II

Morphotype II tracks are documented from the Maphutseng field ichnosite, cast material, historical photographs, and interpretive sketches (Figs 1, 4 and 5; Table 1; S1 Fig; S1 and S2 Tables). At Maphutseng, the high density, (45 tracks preserved on a 90 cm by 65 cm slab), asymmetrical tracks are preserved as epireliefs. Although some of the tracks may be penetrative, it is unlikely the case for the measured tracks as they do not appear to have exaggerated depth differences (all tracks are shallow) and have consistent morphological parameters within a single site and across sites of variable age (Table 1; S1 Fig; S2 Table). Morphotype II tracks preserved at the Maphutseng field ichnosite are small (track length < 5.5 cm), wider than longer (TL/TW of 0.88±0.18), with slender digits which taper to sharp clawed tips. Digital pad impressions are rarely preserved, and when present, they are indistinct (e.g., track 17 digit III; S1 Fig). The tracks are preserved either as discrete digit impressions (where LII<LIV<LIII) or with a merged, V-shaped proximal “heel” where proximal digit IV is offset from the central axis of digit III. The outer digits are widely splayed, with an average total digit divarication of 109°, reaching a maximum of 160°. A single step (tracks 20 and 21) is preserved at Maphutseng, with the tracks oriented parallel to the step midline (S1 Fig). Morphotype II cast materials have comparable morphologies to the field material (average TL/TW of 0.83±0.09 vs 0.88±0.18, DP/TW of 0.41±0.02 vs 0.49±0.16, and II^IV 96° vs 109°; Table 1), though a single larger track is documented (track 39, repository A# 48,2, with TL of 7.2 cm; S1 Fig). Tracks from historical photographs and the interpretive sketches have morphologies and measured parameters comparable to the field and cast material: average TL/TW of 0.86±0.12 vs 0.87±0.16, DP/TW of 0.47±0.09 vs 0.47±0.14 and average II^IV of 105°±14° vs 106°±21°, though a single larger track is documented in the photographs (TL of 11. 74 cm; Table 1; S2 Table). Historical photographs from Maphutseng were matched to tracks preserved in the field (S1 Fig); however active local brick making has destroyed much of the track-bearing surface. It is important to note that tetradactyl Morphotype II tracks are depicted in the interpretive sketches (e.g., track 62) but are not observed in the field material or cast material. Ellenberger [17] noted apparent hallux impressions for *T*. *aviforma* at Maphutseng, and we have matched one of the tracks (his plate 15) to our field material (S1 Fig)–it is our interpretation that in this example the feature close to the heel is not a hallux impression. Morphotype II trackways are exclusively known from the interpretive sketches: most of the tracks have an inward rotation (average -15°), with high pace angulations (average 158°) and stride length up to 10 times the track length (S1 Fig; S3 Table).

The small track lengths, slender digits and wide digit divarication of Morphotype II resembles *Gruipeda* [22], known in the stratigraphic record since the Late Cretaceous. *Gruipeda* is defined as having three forward facing digits (with a fourth backwards facing hallux), interdigit angles less than 70° (II^III and III^IV), with an absence of webbing and, where present, a digital pad formula of 2:2:3:4. *G*. *dominguensis* [22] is small (track length < 5 cm) and wide (TL/TW of 0.7–0.9), and can be preserved as tridactyl or tetradactyl (three forward facing digits with a fourth backward facing hallux). Digit II is the shortest and digit III is the longest (LII < LIV < LIII), with a large total interdigit divarication angle of 90–135° (II^IV). Bipedal *G*. *dominguensis* trackways comprise tracks that are parallel or inwardly rotated relative to the midline, with high pace angulations (150° to 182°) and stride lengths up to 5 times the track length. Morphotype II is rarely preserved with a hallux impression but the forward-facing track morphologies are consistent with *Gruipeda which is* attributed to an avian trackmaker.

When considering conventional TL/TW vs II^IV biplots, Morphotype II is highly variable and coincide with Morphotype II (Fig 5B). The TL/TW and II^IV ranges of Morphotype II are indistinguishable from recently reported Jurassic *Trisauropodiscus* and Cretaceous avian ichnogenera, with minor Morphotype II tracks overlapping with *Anomoepus*. When considering landmark-based principal component analysis which accounts for more of the track morphology, Morphotype II occupies the same morphospace as younger Middle Jurassic *Trisauropodiscus* reported from North Africa and various Cretaceous ichnotaxa attributed to avians (Fig 5C). These morphospace quadrants are most strongly influenced by landmarks 3–8, which capture digit IV ends and digit width; these ichnotaxa are typified by their narrow digits and wide total digit splay. The Morphotype II morphospace only partially overlaps with recent reports of *Trisauropodiscus* from Early Jurassic strata in Lesotho.

The strong resemblance of Morphotype II to established avian ichnogenera and their clear distinction from classical ornithischian and theropod tracks, which is now reinforced by geometric morphometric analyses, supports the original avian affinity of *Trisauropodiscus* originally proposed by [15, 16, 17, 18, 19, 20].

## Discussion

Our reassessment of Ellenberger’s *Trisauropodiscus* material shows that: 1. There are two distinct *Trisauropodiscus* morphotypes and all erected species cannot be synonymized with *Anomoepus*. It is worth noting, that recently reported *Trisauropodiscus* from the Lower [23] (Lesotho) and Upper Jurassic [12] (Morocco) are also considered morphologically distinct from *Anomoepus* by their respective authors, which is supported by our landmark-based principal component analysis (Fig 5C); 2. Our *Trisauropodiscus* Morphotype II has a convincingly avian affinity but is not distinctly avian, as it lacks a well-developed digit III metatarsophalangeal pad and preserves no direct evidence of associated hallux impressions, and; 3. These bird-like *Trisauropodiscus* tracks are known from multiple ichnosites across the Late Triassic to the Early Jurassic of southern Africa (with c. 215.4-Ma-old [29] Morphotype II tracks documented at the Maphutseng field ichnosite).

Basal birds and dinosaurs co-existed in time and their tracks are often preserved on the same ancient tracking surfaces [25, 56]. Bird and non-avian dinosaur tracks have similar morphologies, and although a consensus has not been reached, criteria have been proposed and are commonly used, to distinguish the two [11, 22, 25, 57]. Morphotype II preserves the following bird-like characteristics: 1) an overall resemblance to modern bird tracks; 2) small size (length < 7.5 cm); 3) wider than long tracks; 4) slender digits with indistinct pad impressions; 5) large total digit divarication angles (typically II^IV > 100°), and 6) an inward rotation of tracks relative to the trackway midlines. Additional site-wide features often considered to strengthen an avian interpretation [22, 25], which are observed for Morphotype II track-bearing palaeosurfaces, include: 1) a high track density; 2) a lack of preferred track orientation, and 3) an association with invertebrate traces. However, these characteristics are not exclusive to avians and are also known from dinosaur tracksites.

Identifying a specific tracemaker for these Late Triassic to Early Jurassic footprints is challenging, despite their morphological characteristics, such as slender digits and large total digit divarication angles, suggesting a bird-like foot. For example, more recently, some authors have suggested a small, gracile theropod tracemaker such as coelurosaurs (including Paraves [26]) for *Trisauropodiscus* while others attribute the tracks to ornithomimipodids (ornithomimids [12]). Given the evolutionary patterns among tetrapods, it is also possible that unrelated Mesozoic groups, such as tridactyl archosaurs, developed convergent pedal morphology leading to a bird-like foot e.g., *Poposaurus gracilis* resembles a theropod track [58]. Considering the spatiotemporal distribution of the track-bearing surfaces (Fig 1), three subgroups of tridactyl archosaurs—ornithischians, theropods, and basal birds—could be the putative tracemakers. However, it is also possible that an unknown facultatively tridactyl archosaur could have potentially produced the tracks. Ornithischian and theropod body fossils are present in the Mesozoic rock record of southern African, and while the former is well-documented [59] the latter is scarce [60, 61], with both regularly being ascribed as the trackmakers of various tridactyl tracks in the region [23, 31, 43, 44, 45, 62 – 68]. The morphological features (e.g., digit width, total digit divarication angle) of the bird-like *Trisauropodiscus* are incompatible with these local, contemporaneous, similarly sized tridactyl tracks [23, 64] and it is unlikely they were registered by the same tracemaker.

Globally, the only Late Triassic body fossil specimen posited to be bird-like is Protoavis [13], but this assessment is based on ambiguous material [3, 14]. Neornithes first appear in the fossil record in the latest Cretaceous, and their presence is contemporaneously noted in both the skeletal [69] and trace fossil records [56]. Therefore, it is likely that Morphotype II tracks were made by a yet-to-be-found tridactyl archosaur. That these tracks of southern Africa, dating to the Late Triassic, so strongly resemble Cenozoic and modern bird tracks substantiates the converging pedal morphology of Late Mesozoic archosaurs [2, 8] and firmly shows that the origin of bird-like foot morphology is at least ~210 million years old [29].

## Supporting information

S1 Fig Photographs, interpretive outlines and false -colour depth maps of Late Triassic–Early Jurassic Elliot Formation *Trisauropodiscus* field material, cast material (housed at Morija Museum and Archives and Université de Montpellier) and published illustrations (12, 13) considered in this study. Based on distinct morphological characteristics, the *Trisauropodiscus* tracks can be subdivided into two distinct Morphotypes (I and II, see S2 Table). Published illustrations and photographs are included under a CC BY license with permission from the GSSA and Palaeovertebrata, original copyright [1970 and 1974, respectively].
(PDF)

S2 Fig Morphological comparison of southern African *Trisauropodiscus* morphotypes with established ichnogenera attributed to avian, ornithischian and theropod trackmakers (see Fig 3B). Corresponding track numbers are included (see S1 Fig).
(JPG)

S1 Table *Trisauropodiscus* specimens (published illustrations and cast material collected by Paul Ellenberger) in the Elliot Formation (main Karoo Basin) from four different sites in Lesotho. For location map, see Fig 2; and for additional details see S1 Fig, S2 Table. For 3D photogrammetric modelling data, see supplementary material here: https://figshare.com/s/37f1fc1924aa51828d0e) Abbreviations: UoM–Université de Montpellier, MM&A–Morija Museum and Archives, lEF–lower Elliot Formation, uEF–upper Elliot Formation. Published illustrations are included under a CC BY license with permission from the GSSA and Palaeovertebrata, original copyright [1970 and 1974, respectively].
(DOCX)

S2 Table Track measurement data of *Trisauropodiscus* field material, cast material and published illustrations considered in this study. Blank fields indicate that the specific parameter, defined in Fig 1, could not be measured due to the absence of the necessary feature or unclear track morphology. For field photographs, cast material, published illustrations and historical photographs of the tracks, see S1 Fig; for location map, see Fig 2; Abbreviations: TL–Track length, TW–Track width, II^IV–total digit divarication, lEF–lower Elliot Formation, uEF–upper Elliot Formation.
(XLSX)

S3 Table Trackway measurement data for *Trisauropodiscus* cast material and published illustrations considered in this study. Blank fields indicate that the specific parameter, defined in Fig 1, could not be measured due to unclear track or trackway morphology. For cast material and published illustration trackways, see S1 Fig; for location map, see Fig 2.
(XLSX)

S4 Table Summary statistics of track orientation data. Track orientation is measured from field material, cast material and historical photographs. Abbreviations: Map–Maphutseng. See Fig 2 for site localities.
(XLSX)

S5 Table Detailed track orientation data considering >150 tracks from field material, cast material and historical photographs. Abbreviations: Map–Maphutseng. See Fig 2 for site localities.
(XLSX)
