# Supplementary figures and images for: The oldest fossil bird-like footprints from the upper Triassic of southern Africa
Source: PLoS One. 2023 Nov 29;18(11):e0293021. doi: 10.1371/journal.pone.0293021 (PMC10686444; doi:10.1371/journal.pone.0293021)

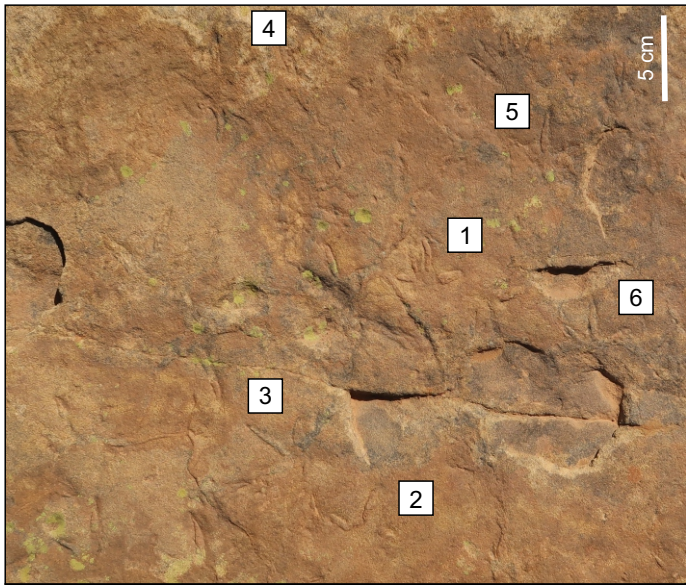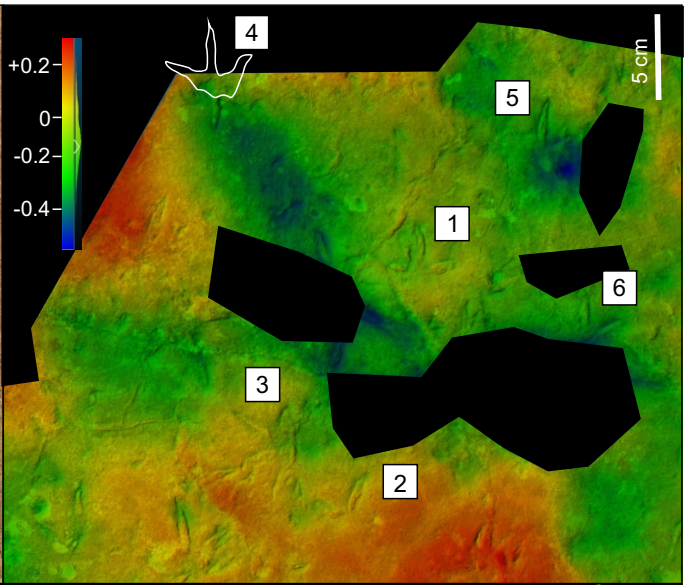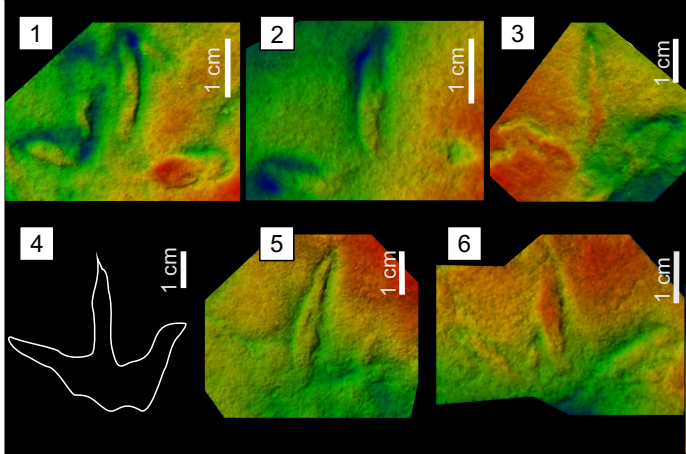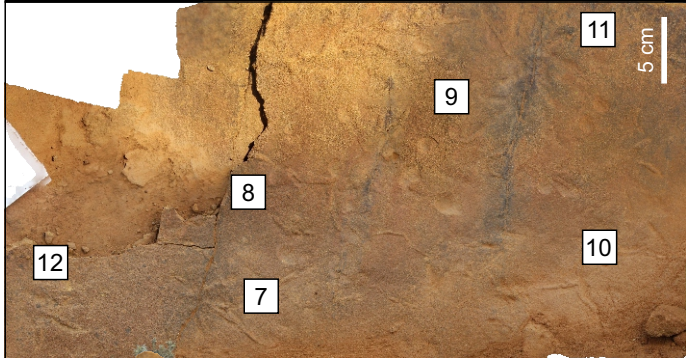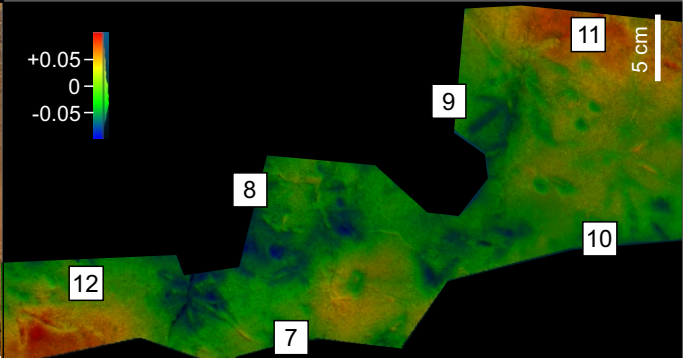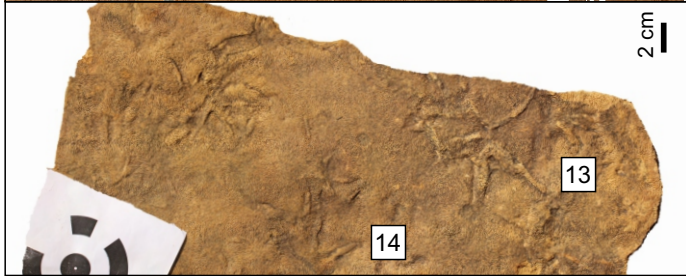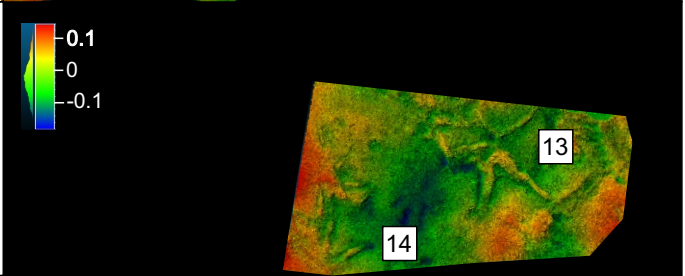

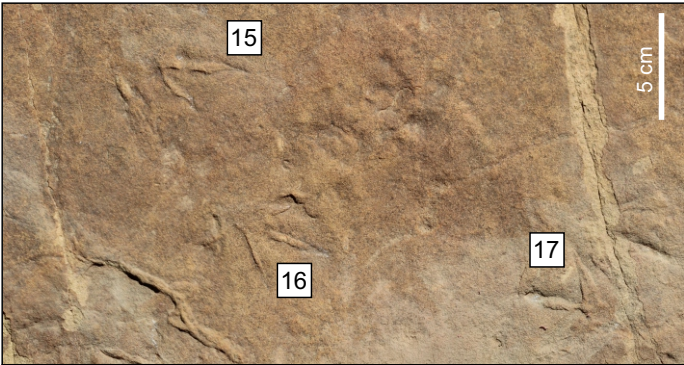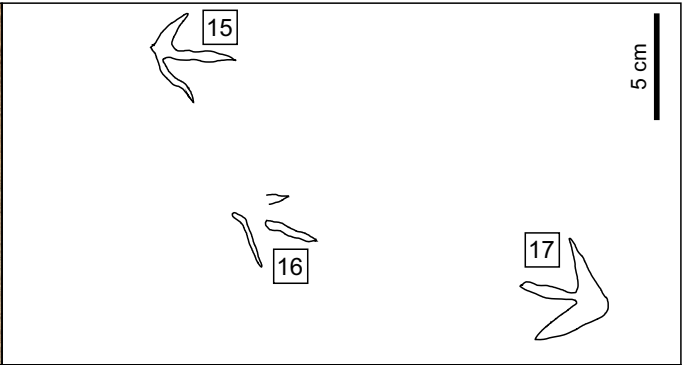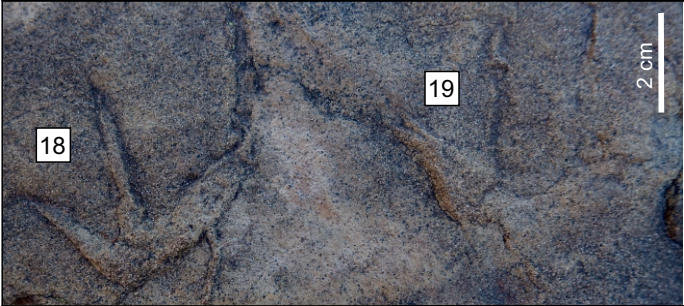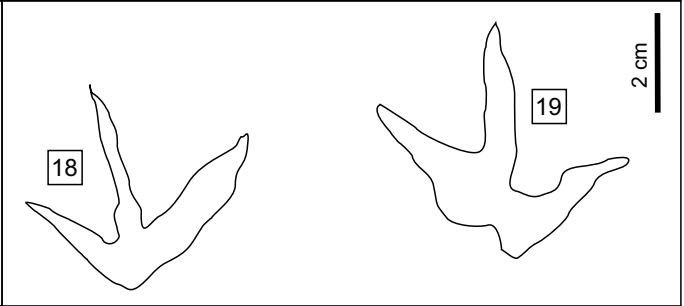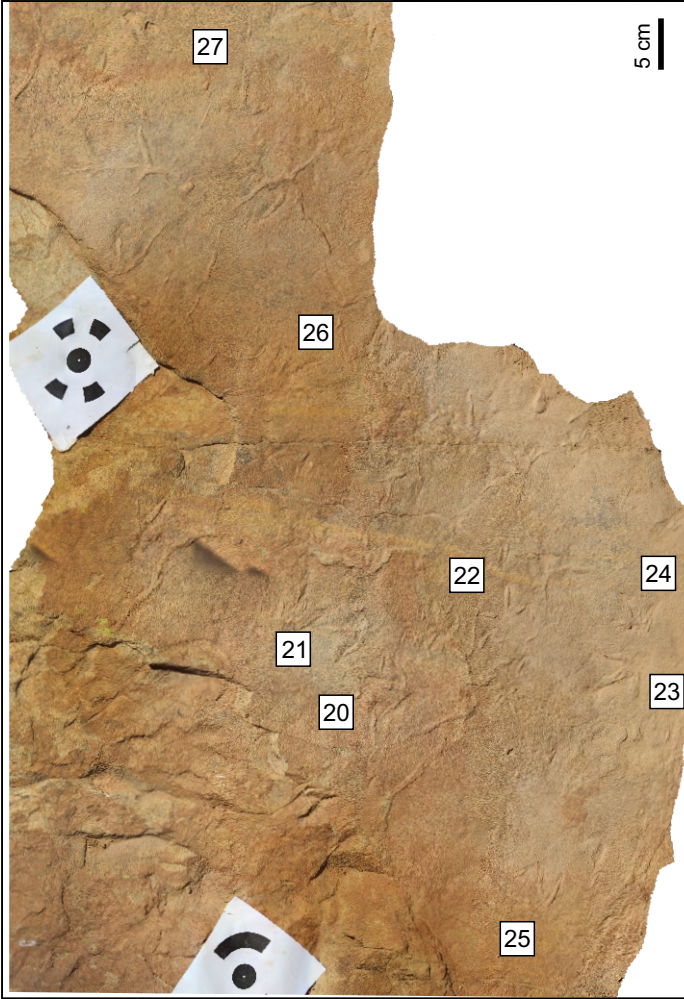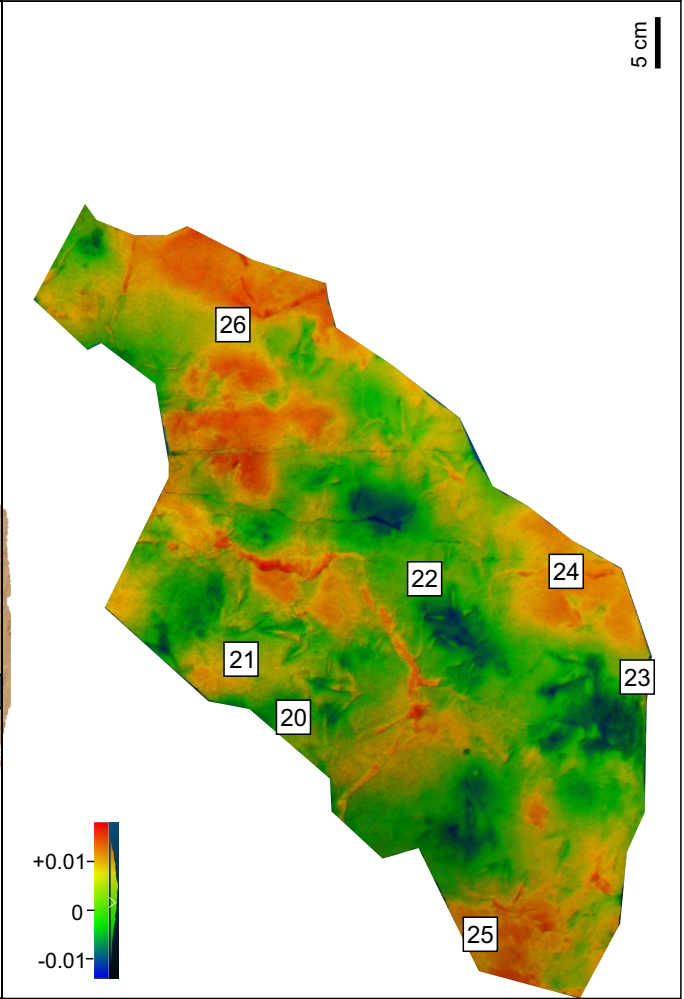

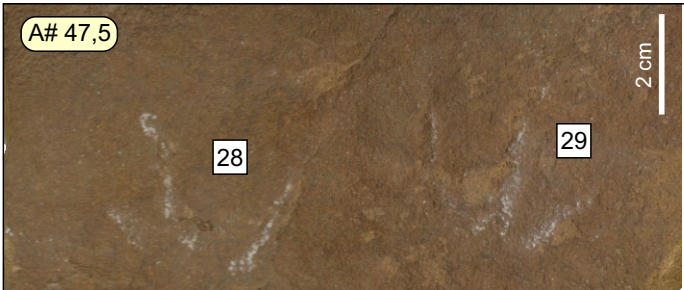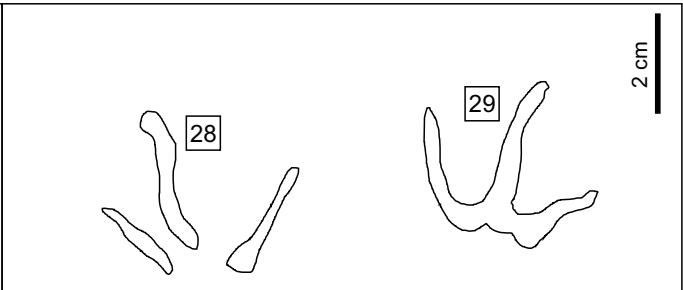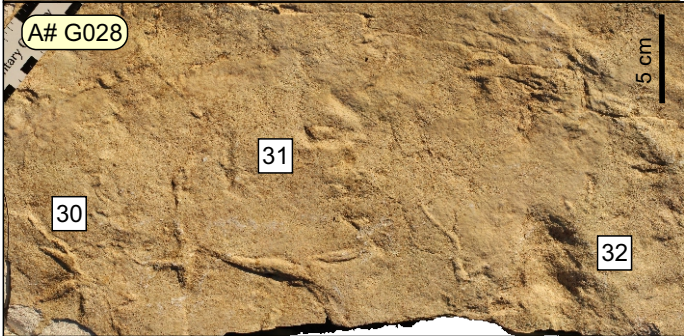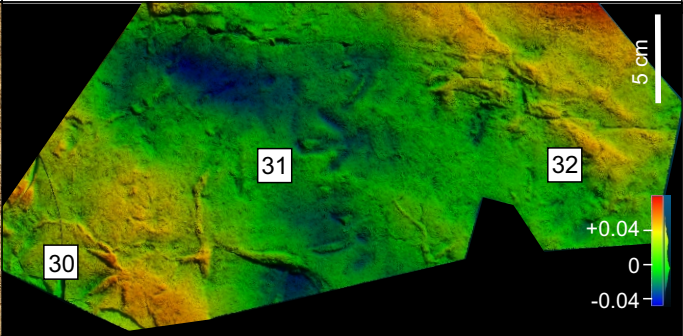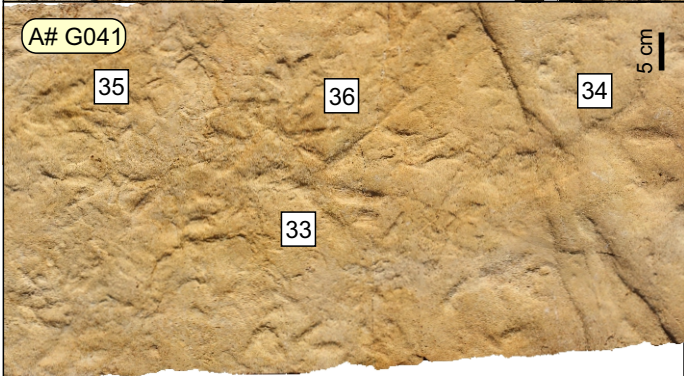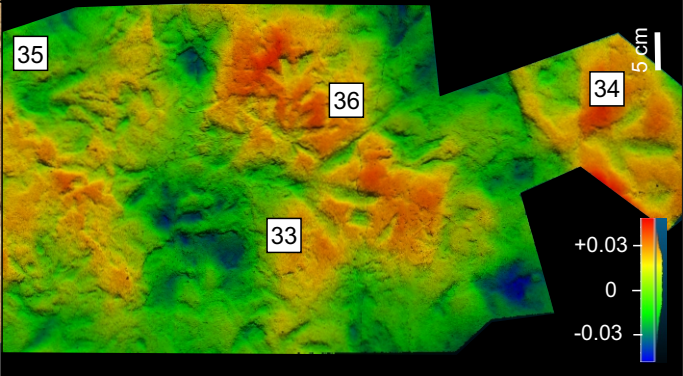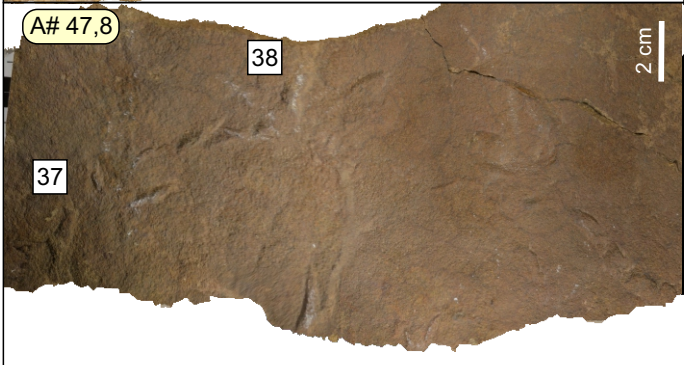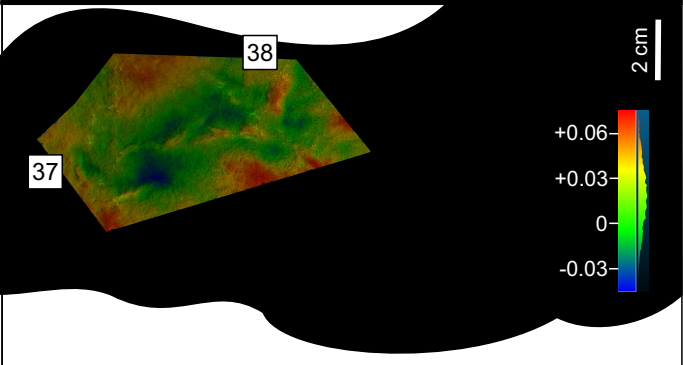

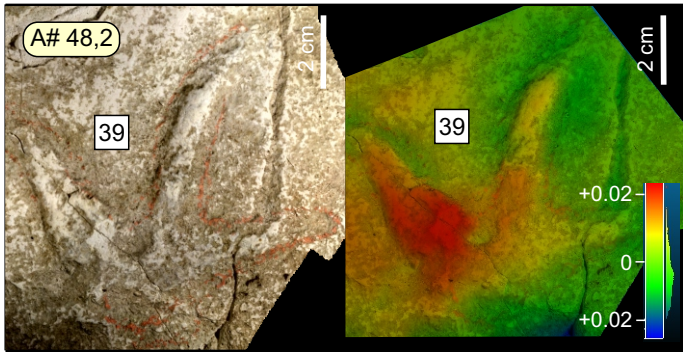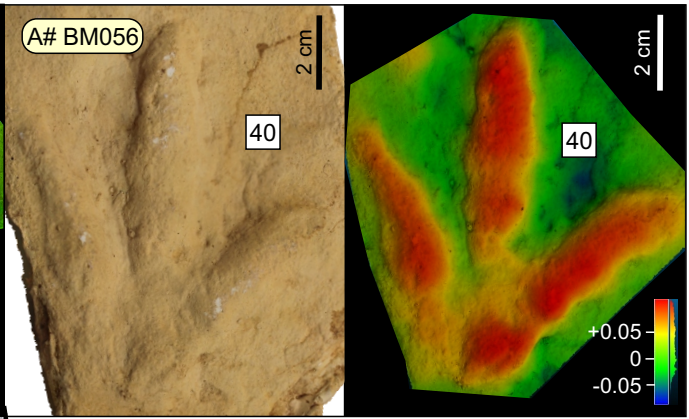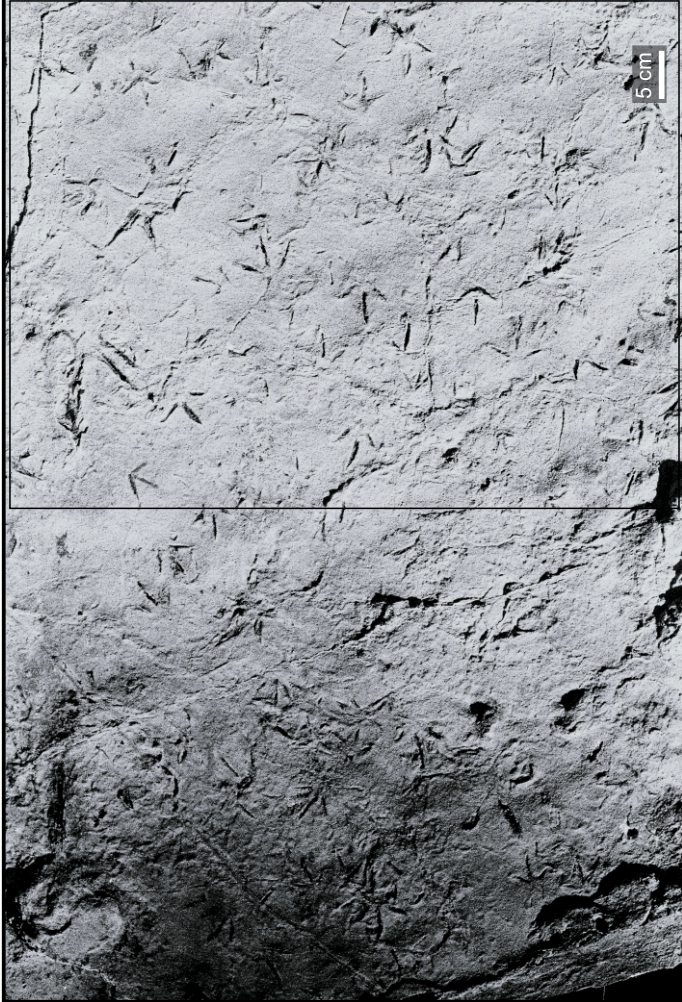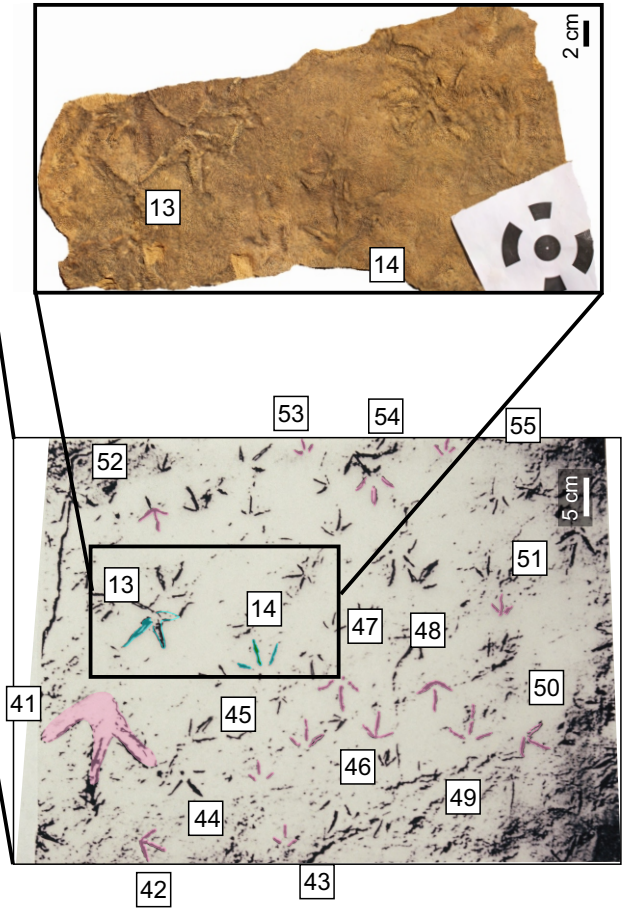

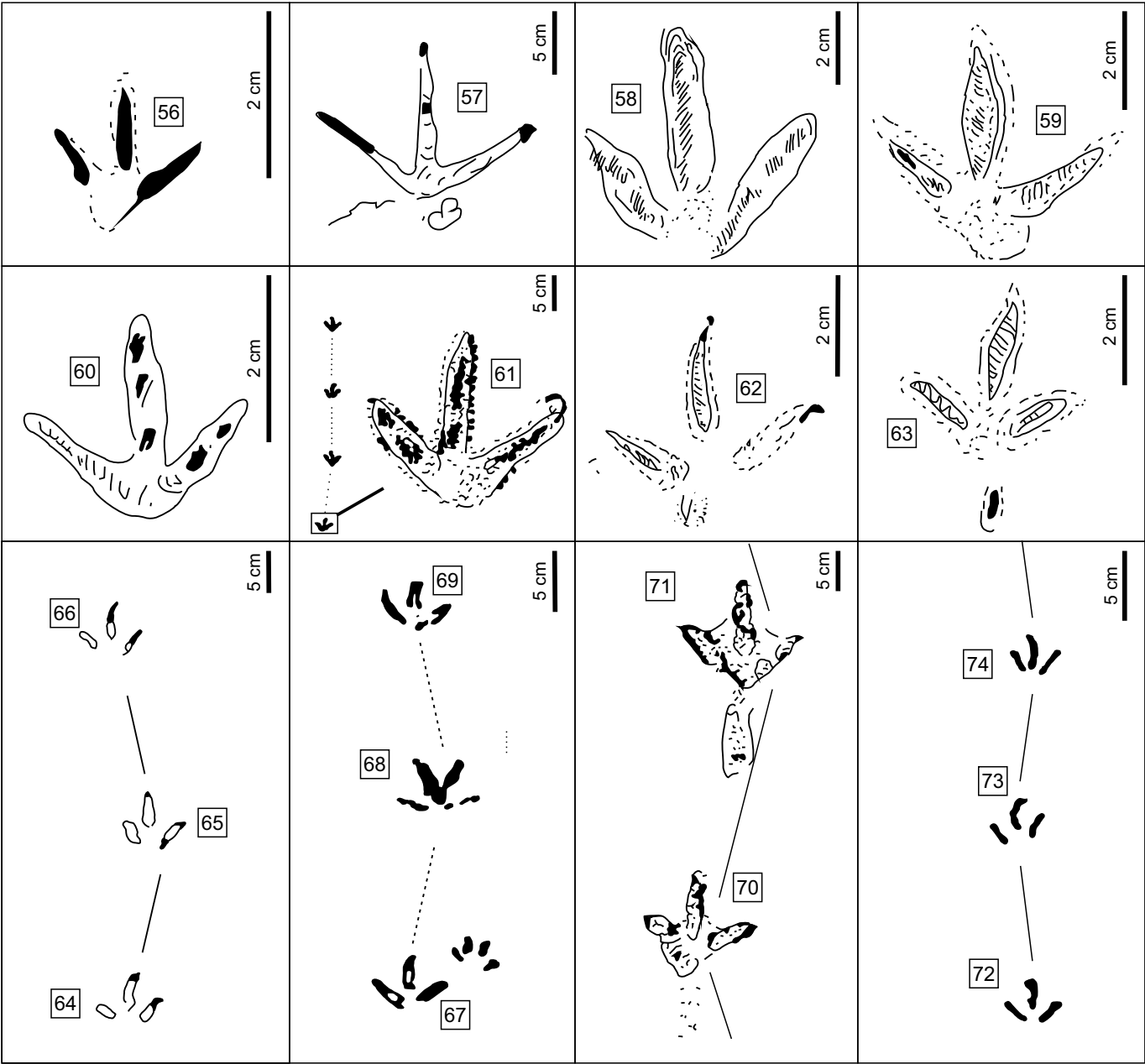

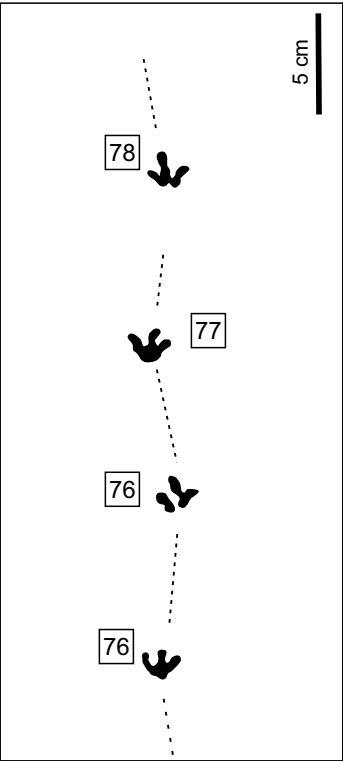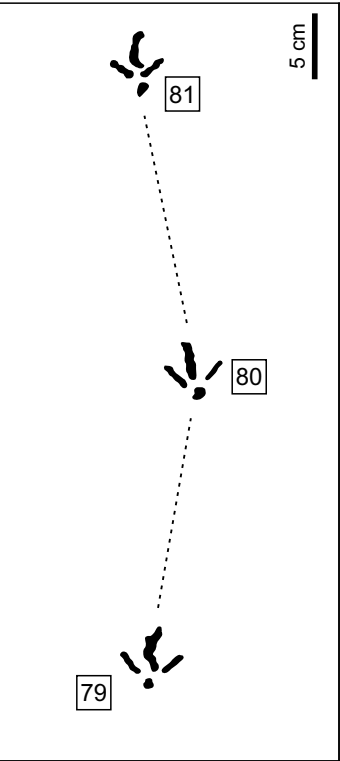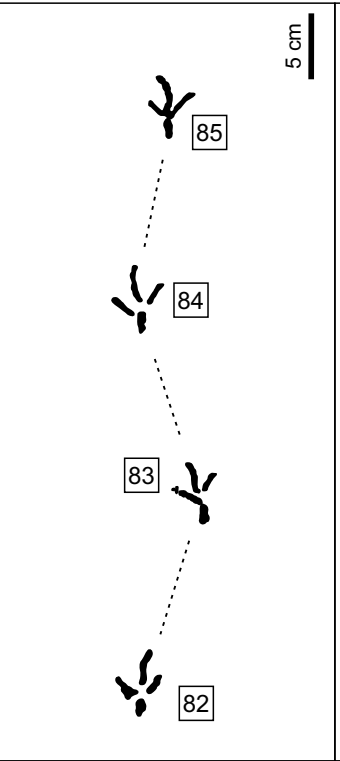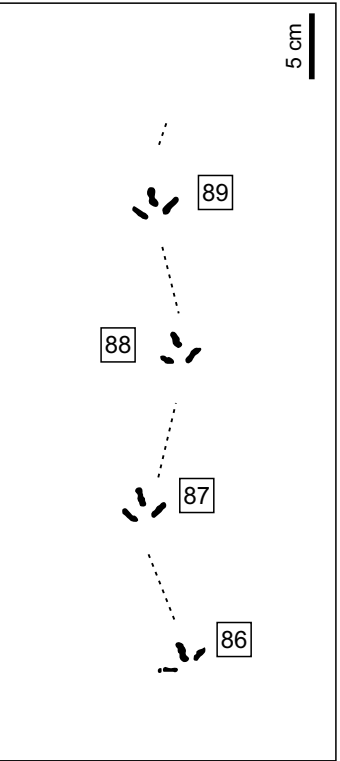

Supplement: S1 Fig — Based on distinct morphological characteristics, the Trisauropodiscus tracks can be subdivided into two distinct Morphotypes (I and II, see S2 Table). Published illustrations and photographs are included under a CC BY license with permission from the GSSA and Palaeovertebrata, original copyright [1970 and 1974, respectively]. (PDF) [file pone.0293021.s001.pdf]

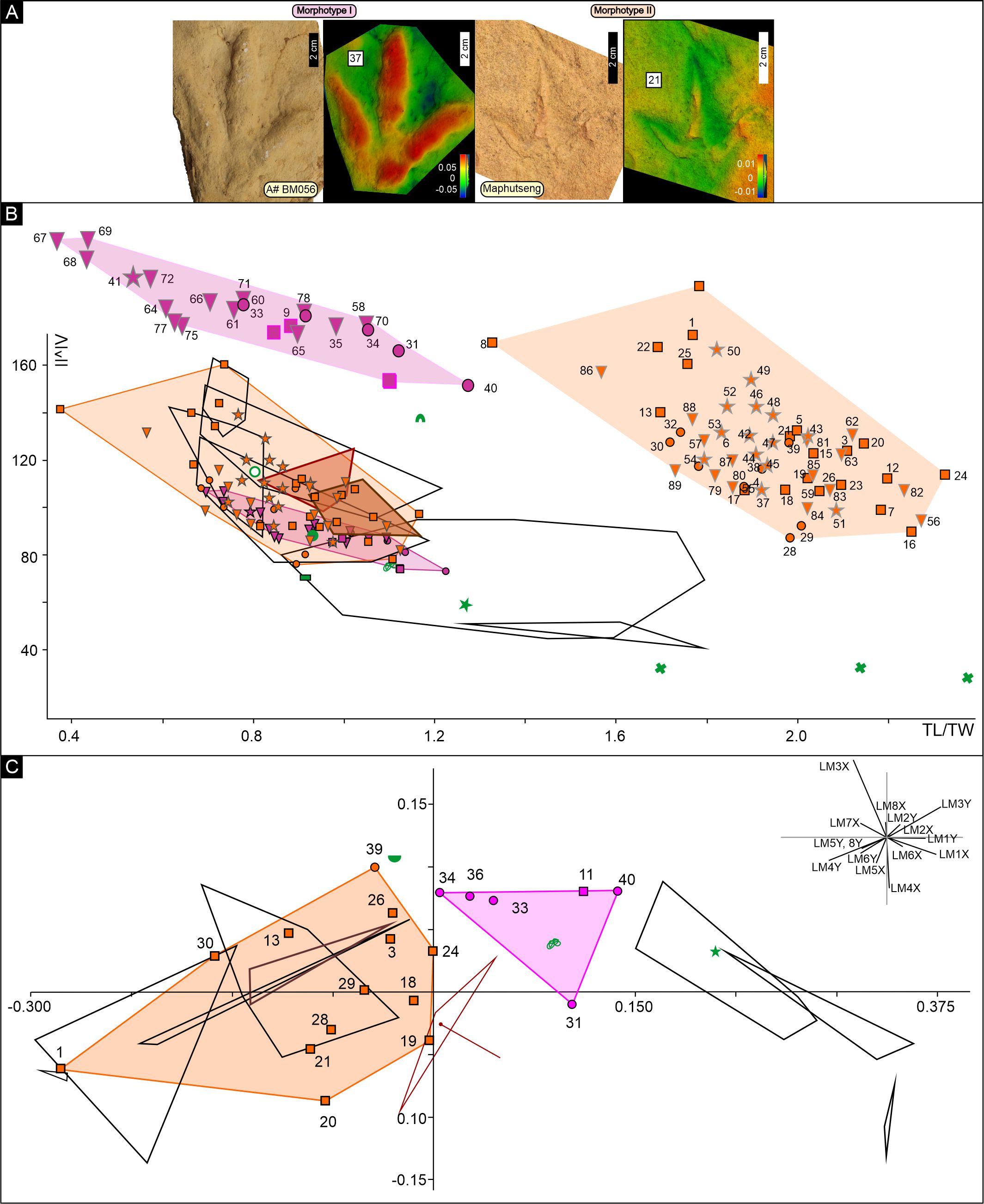

Supplement: S2 Fig — Corresponding track numbers are included (see S1 Fig). (JPG) [file pone.0293021.s002.jpg]
