# Supplementary material for: The oldest fossil bird-like footprints from the upper Triassic of southern Africa
Source: PLoS One. 2023 Nov 29;18(11):e0293021. doi: 10.1371/journal.pone.0293021 (PMC10686444; doi:10.1371/journal.pone.0293021)
Supplement: S1 Table — For location map, see Fig 2; and for additional details see S1 Fig, S2 Table. For 3D photogrammetric modelling data, see supplementary material here: https://figshare.com/s/37f1fc1924aa51828d0e) Abbreviations: UoM–Université de Montpellier, MM&A–Morija Museum and Archives, lEF–lower Elliot Formation, uEF–upper Elliot Formation. Published illustrations are included under a CC BY license with permission from the GSSA and Palaeovertebrata, original copyright [1970 and 1974, respectively]. (DOCX) [file pone.0293021.s003.docx]

| **Ichnotaxa** | **Location** | **Ellenberger’s biozone #** | **Refined lithostratigraphic zone** | **Maximum depositional age in Ma (see 33)** | **Primary reference with figure number** | **Morphotype** | **Accession number** | **Current location** |
| --- | --- | --- | --- | --- | --- | --- | --- | --- |
| *Trisauropodiscus aviforma* | Maphutseng (Majakaneng) | A/4 | middle lEF | <215.4, Norian | **Published illustrations**  Ellenberger 1970, **1972**; fig. 47A–E; Plate [XV](https://www.dropbox.com/s/p0gs6t6kar6lcj8/Bordy3811-R1-15-16.JPG?dl=0), XVII; 1974: Plate XVIII  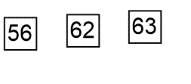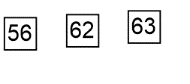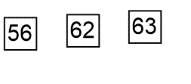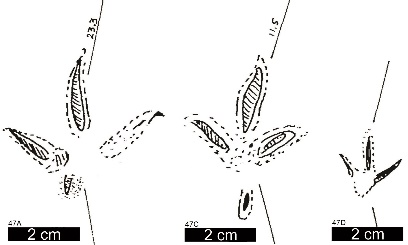  **Cast material**  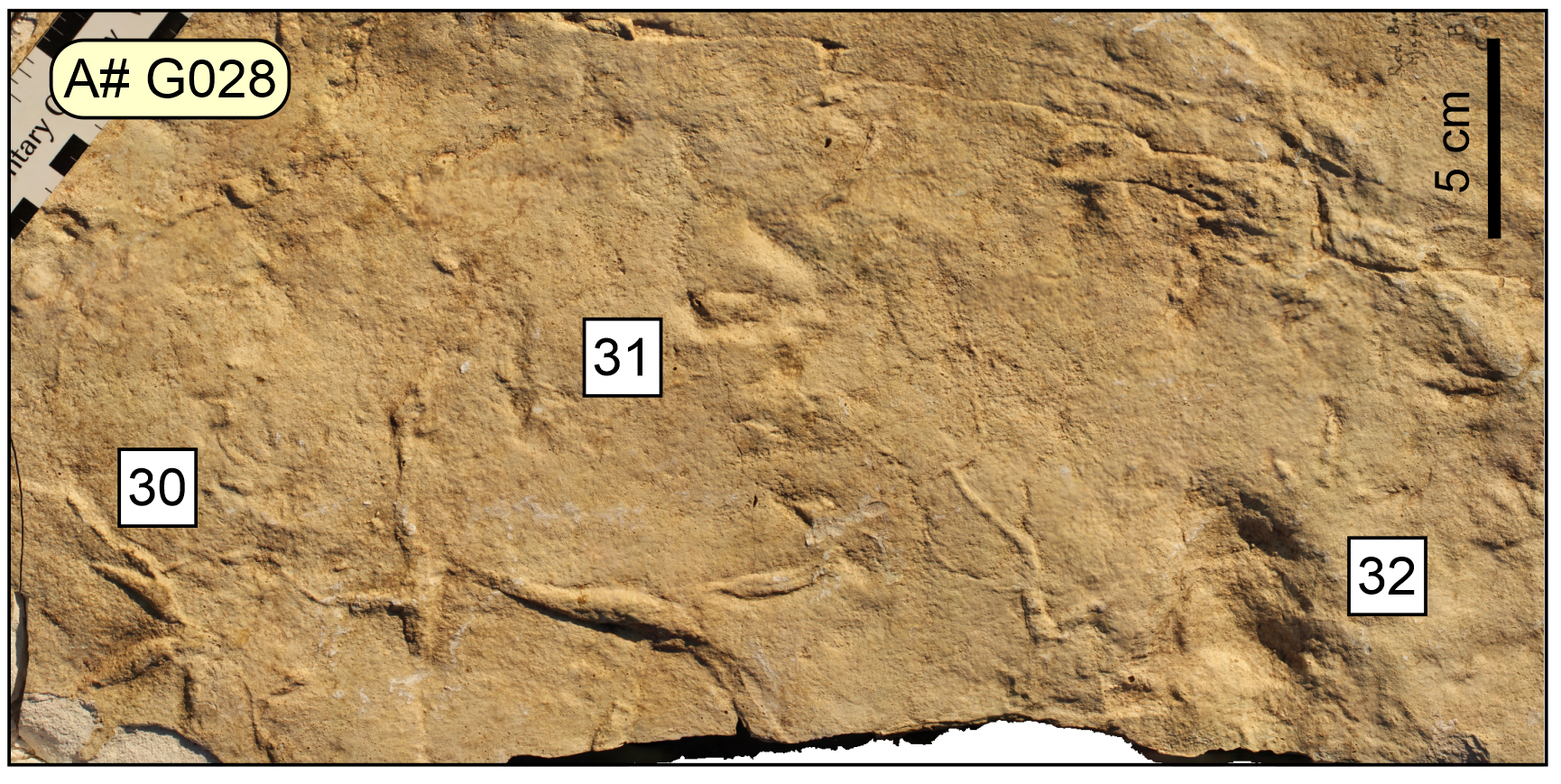  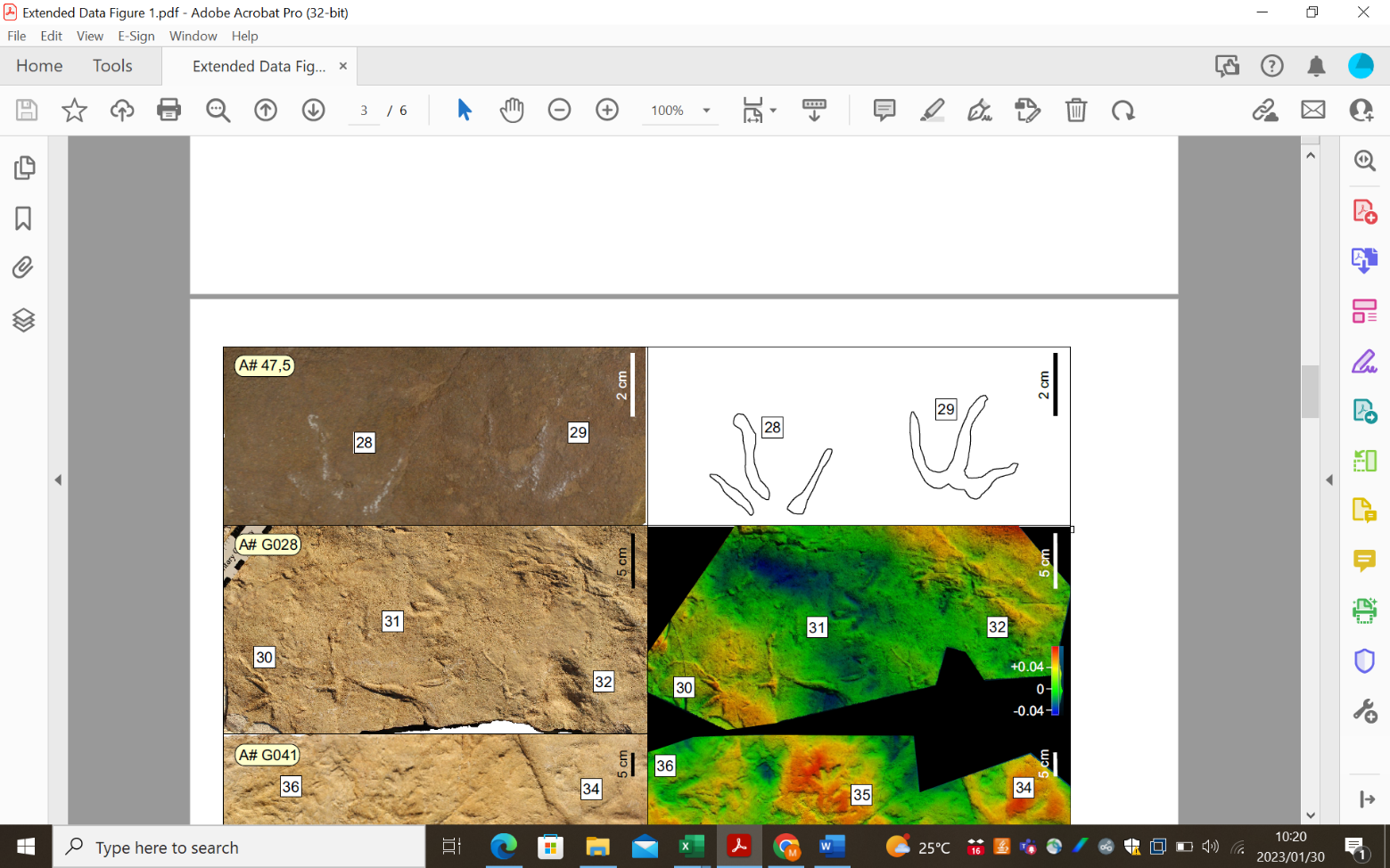  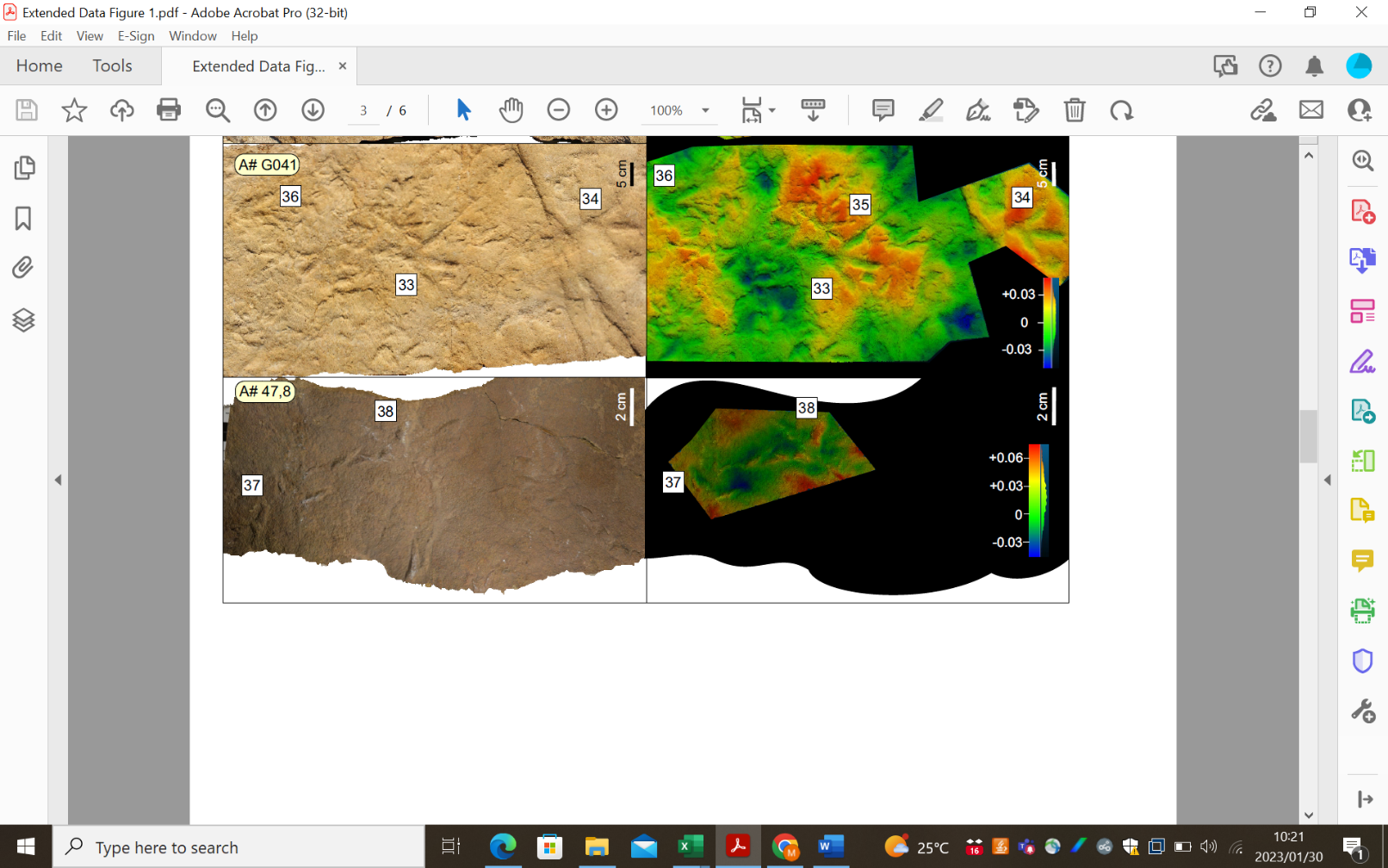 | II | LES 047_1–9 @UoM  G028 @MM&A | UoM, field |
| *Trisauropodiscus superaviforma* | Maphutseng  (Majakaneng) | A/4 | middle lEF | <215.4, Norian | 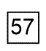Ellenberger 1970, **1972**; fig. 48, Plate [XV](https://www.dropbox.com/s/p0gs6t6kar6lcj8/Bordy3811-R1-15-16.JPG?dl=0) 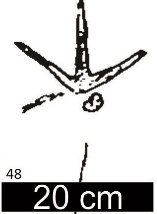 | I | LES 048_1 UoM | (lost from UoM) |
|  | Leribe | A/4 | lowermost lEF | <216.4, Norian | Not illustrated before this current study  **Cast material**  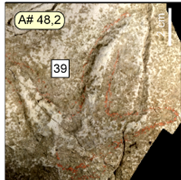 | I | LES 048_2, LES 048_3 @UoM  Note LES08,3 is An inverted replica of 048,2 | UoM |
| *Trisauropodiscus galliforma* | Thejane  (Maphutseng) | A/6 | lowermost uEF | <200, Hettangian-early Sinemurian | 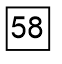Ellenberger 1970**, 1972**; fig. 55, Plate XVII 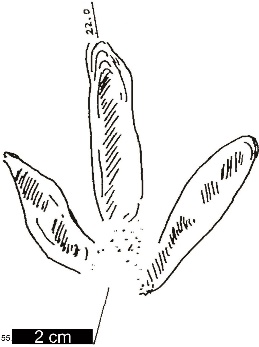  “includes 6 identical incomplete tracks, well-defined including track A with 3 successive steps, in association with T. *phasianiforma*, *T. levis* and *T. popompoi*” | I | LES065 @UoM | (lost from UoM) |
| *Trisauropodiscus phasianiforma* | Thejane  (Maphutseng) | A/6 | lowermost uEF | <200, Hettangian-early Sinemurian | 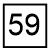Ellenberger 1970, **1972**; fig. 56, Plate XVII 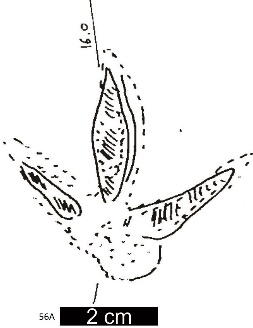 | II | LES066 @UoM | (lost from UoM) |
| *Trisauropodiscus levis* | Thejane  (Maphutseng) | A/6 | lowermost uEF | <200, Hettangian-early Sinemurian | 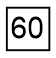Ellenberger 1970, **1972**; fig. 57, Plate XVII 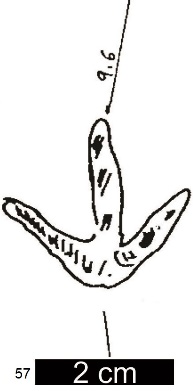 | I | LES067 @UoM | (lost from UoM) |
| *Trisauropodiscus aviforma* | Maphutseng (Majakaneng) | A/4 | middle lEF | <215.4, Norian | Ellenberger, 1970, 1972; fig 47 A-E; planche XVI  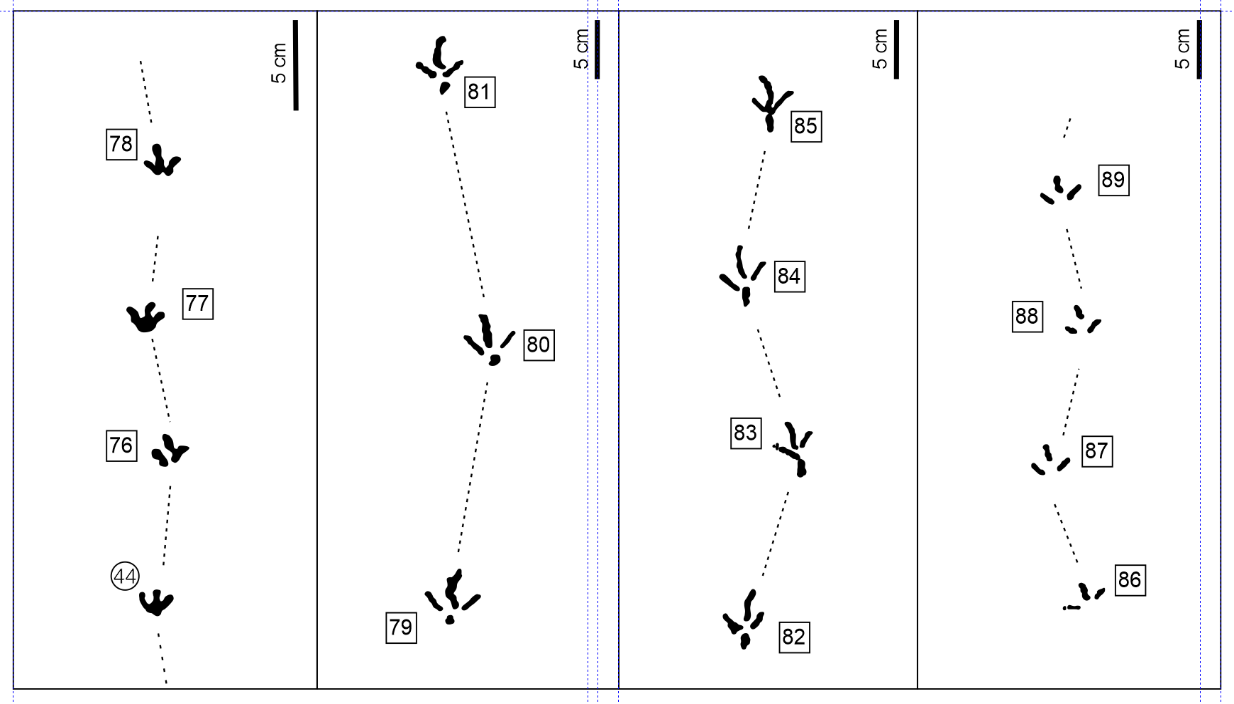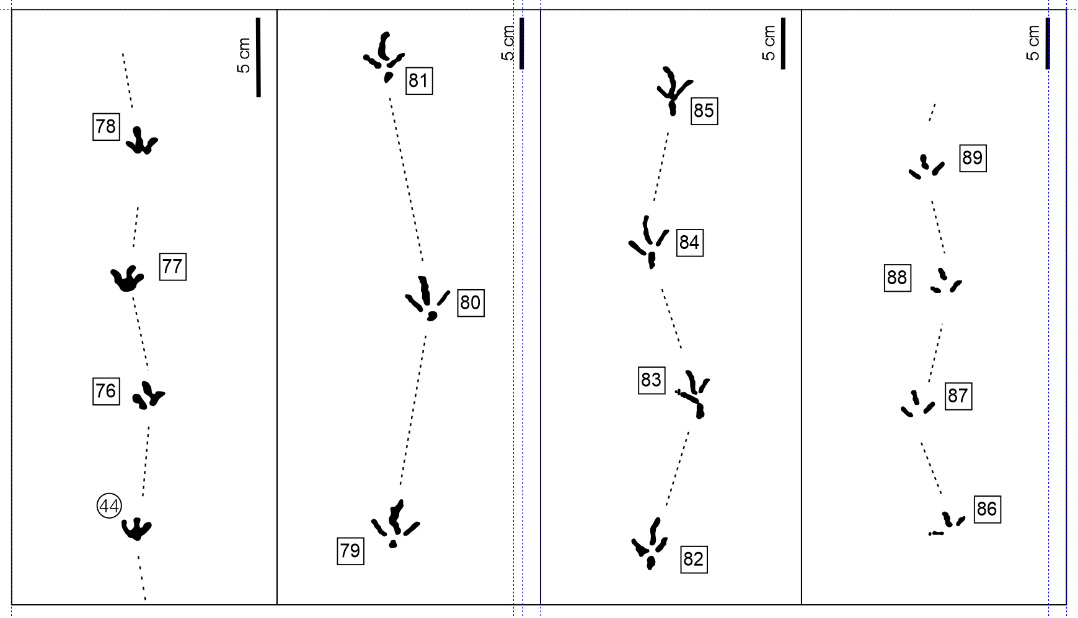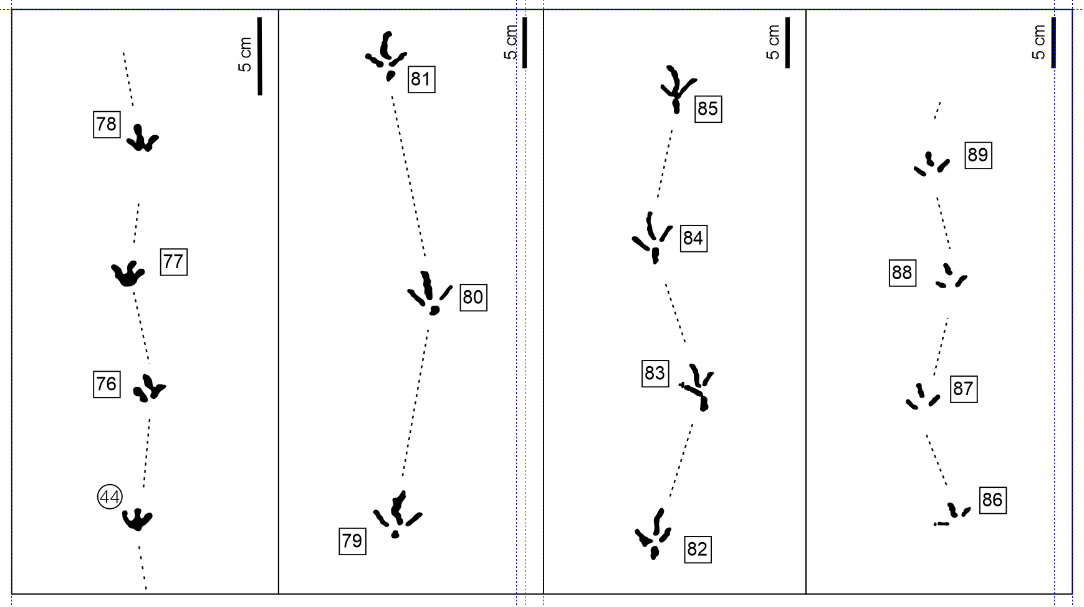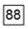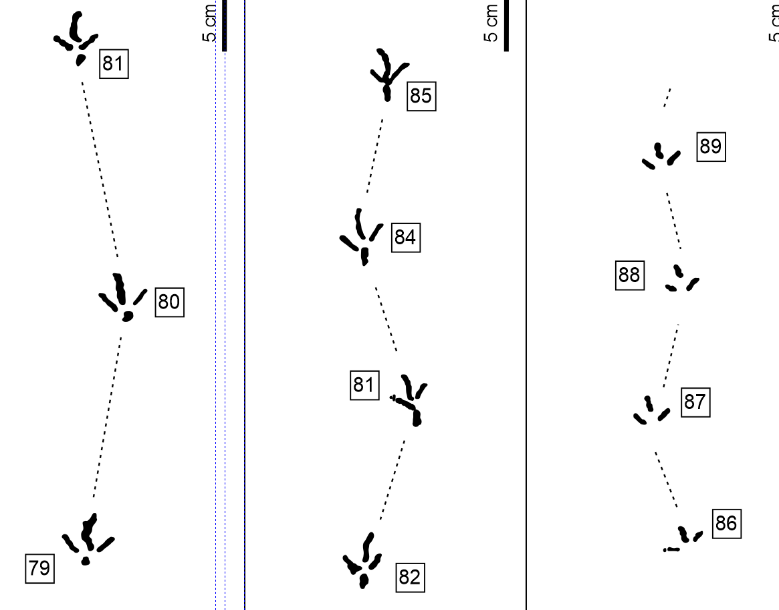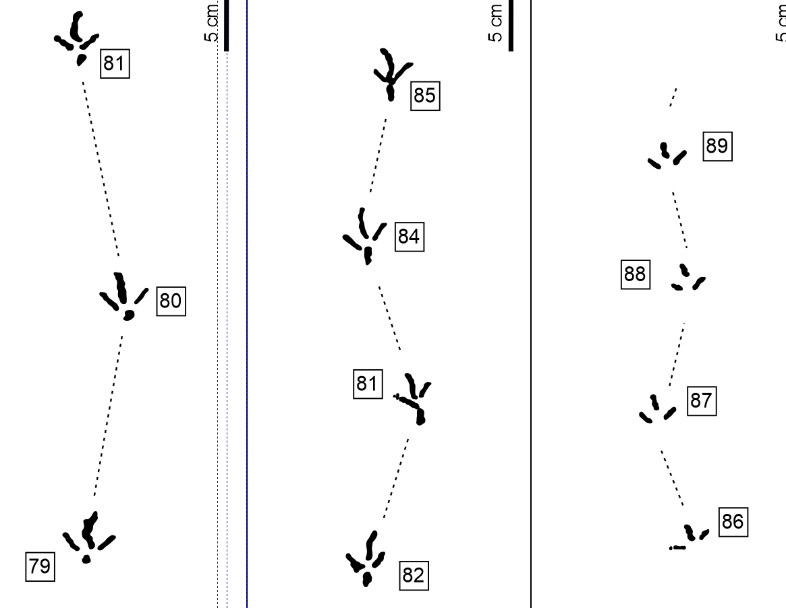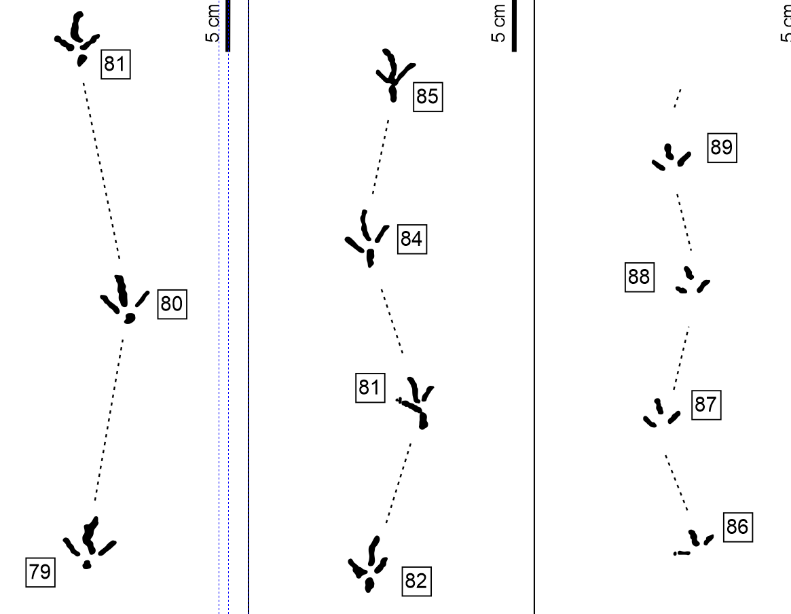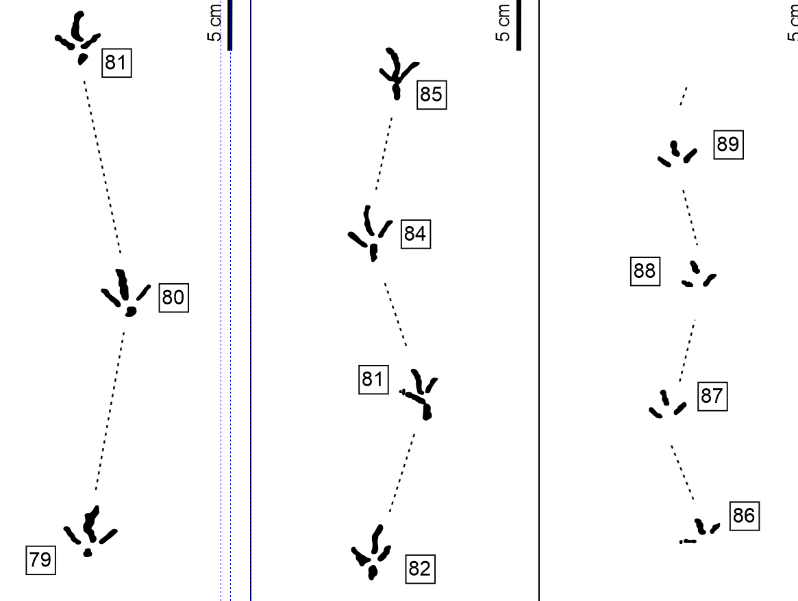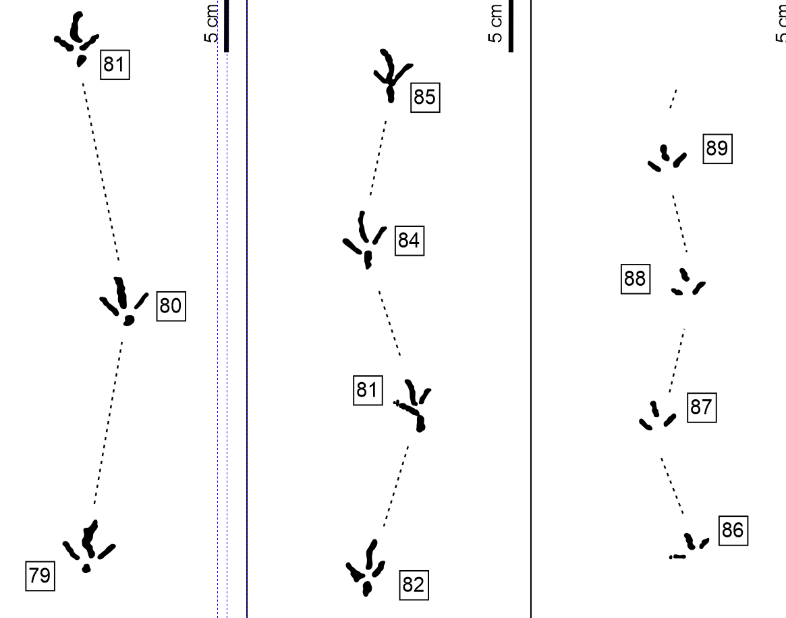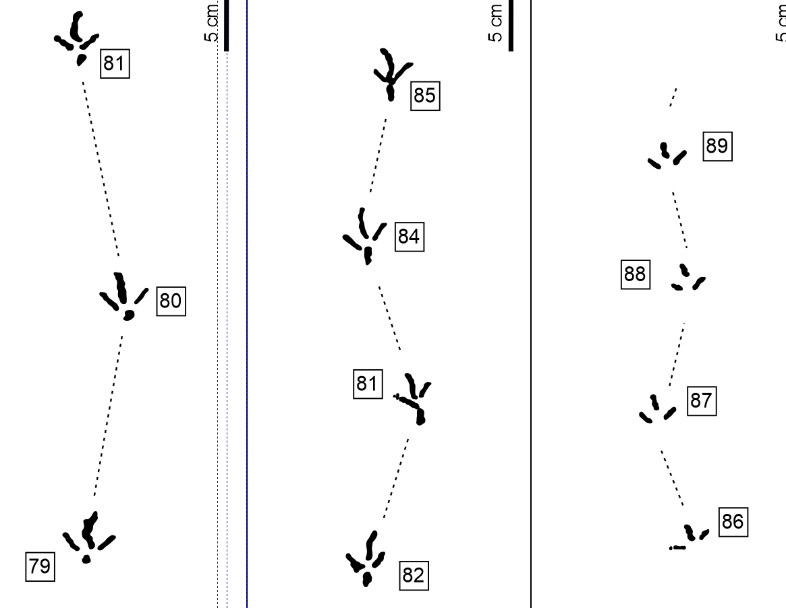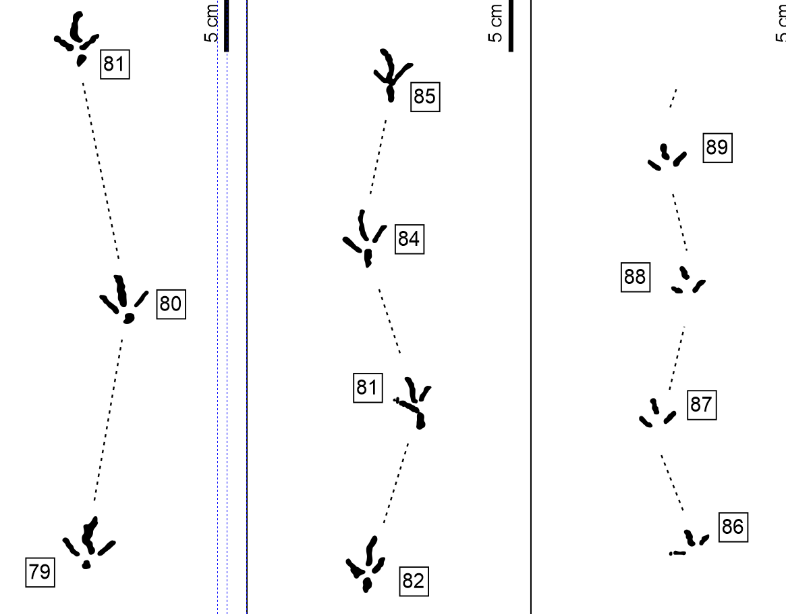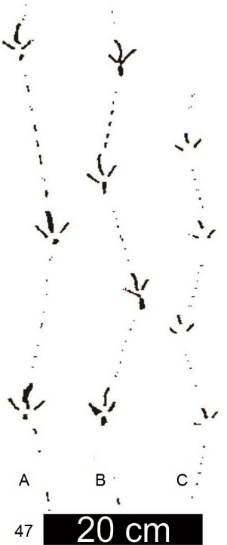  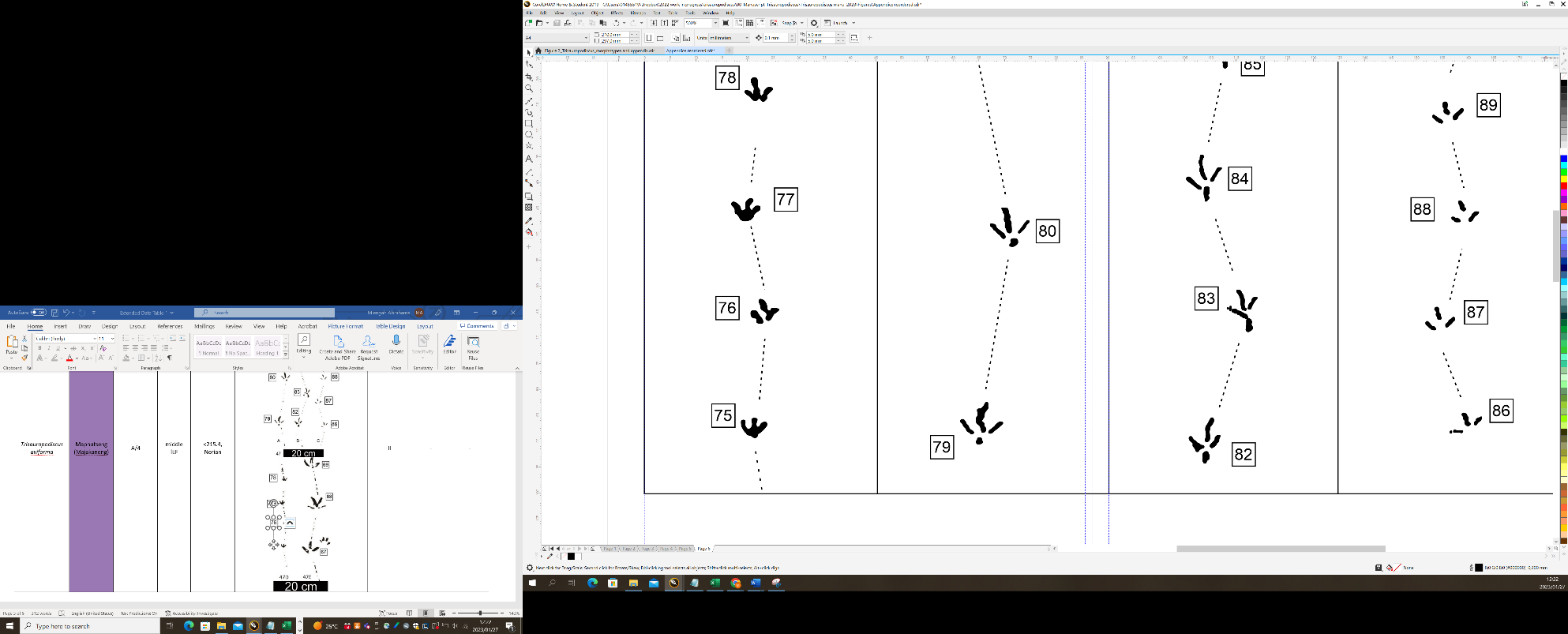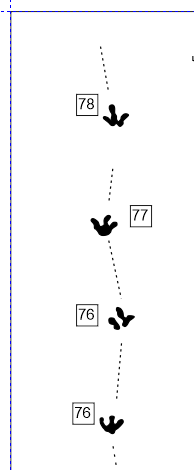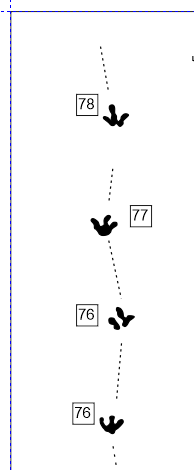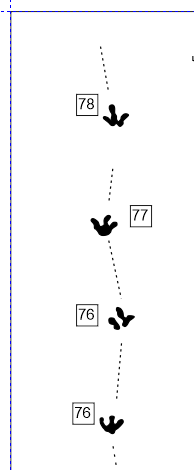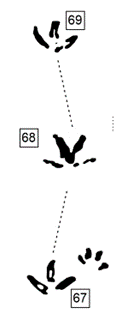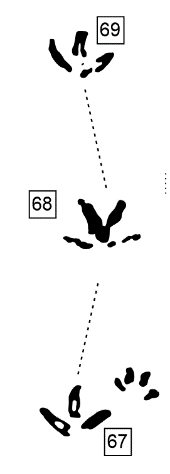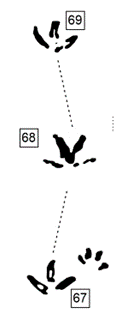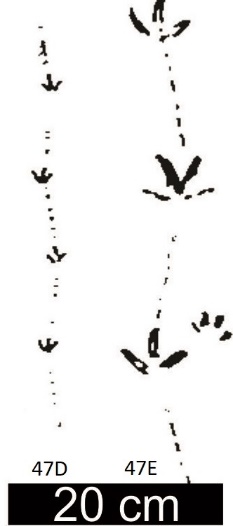 | II | - | - |
| *Trisauropodiscus*  *galliforma* | Thejane | A/6 | lowermost uEF | <200, Hettangian-early Sinemurian | Ellenberger, 1970; 1972; fig. 55; planche XVII  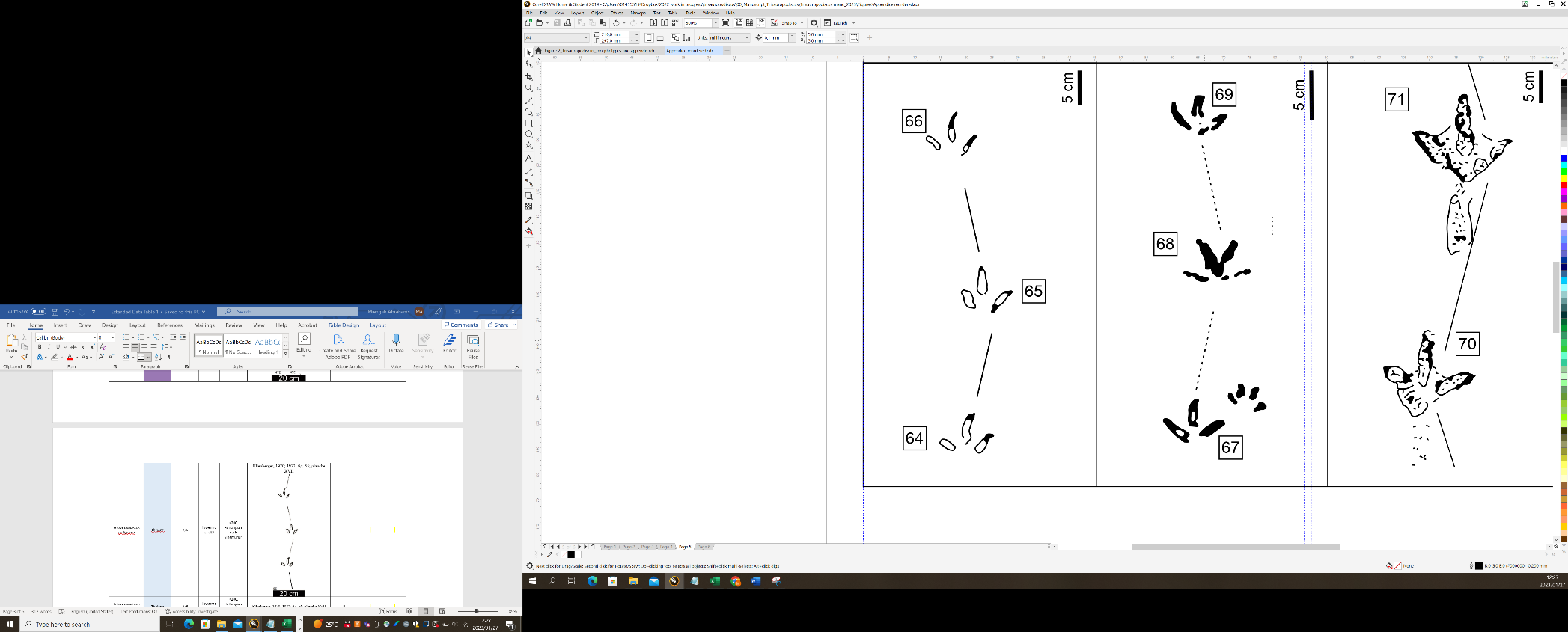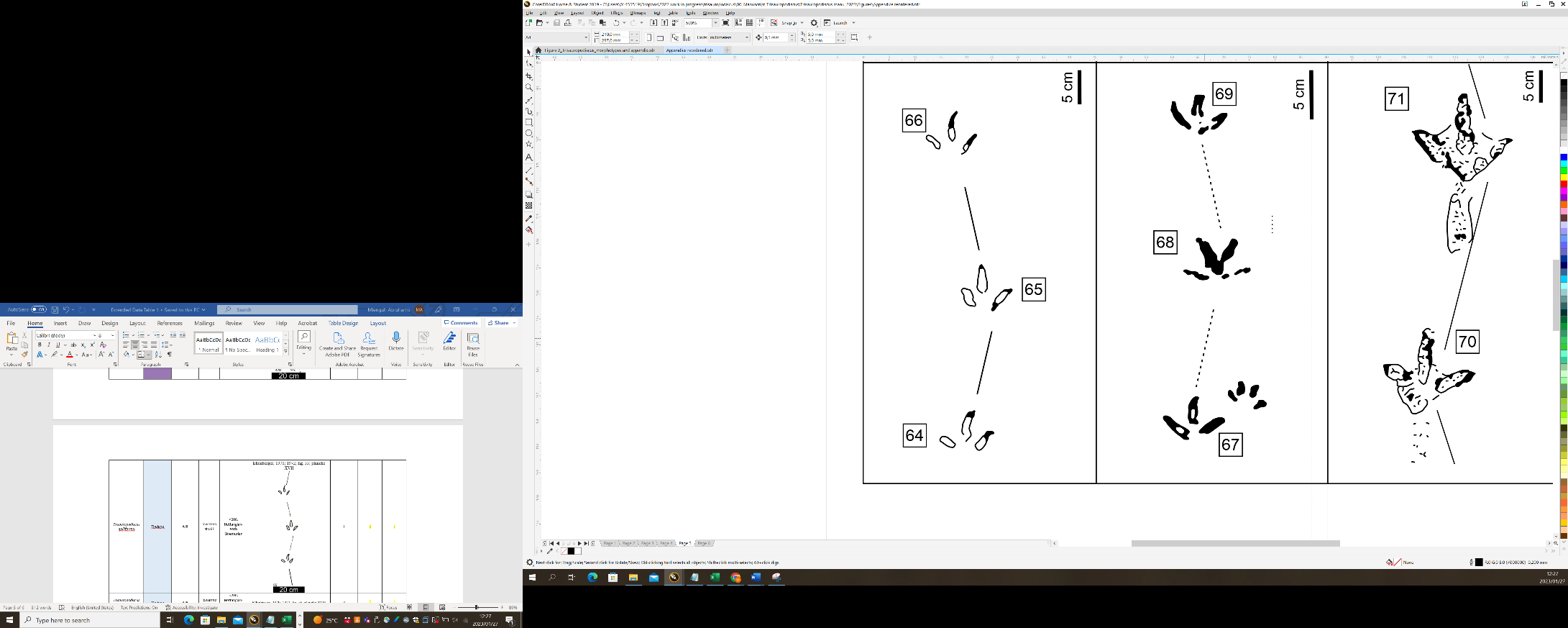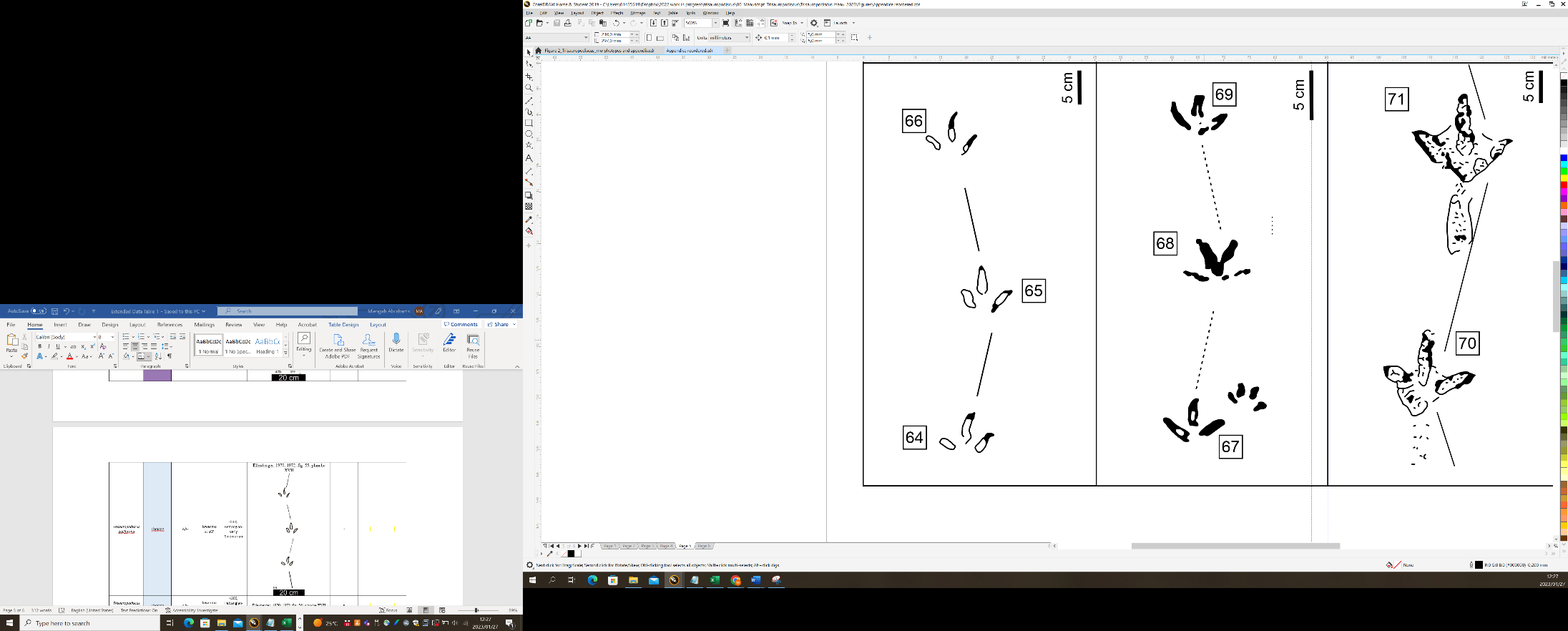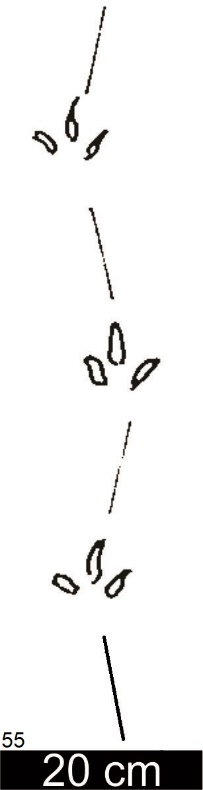 | I | - | - |
| *Trisauropodiscus*  *phasianiforma* | Thejane | A/6 | lowermost uEF | <200, Hettangian-early Sinemurian | Ellenberger, 1970; 1972; fig. 56; planche XVII  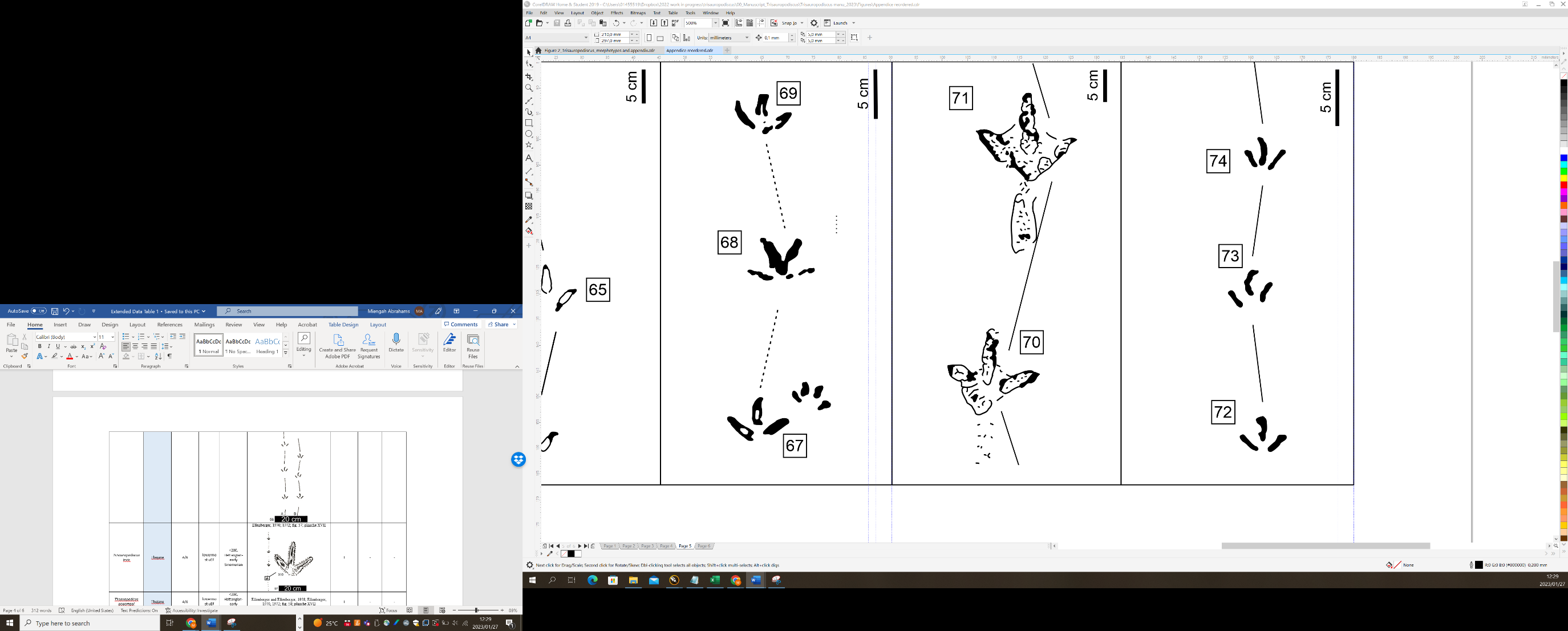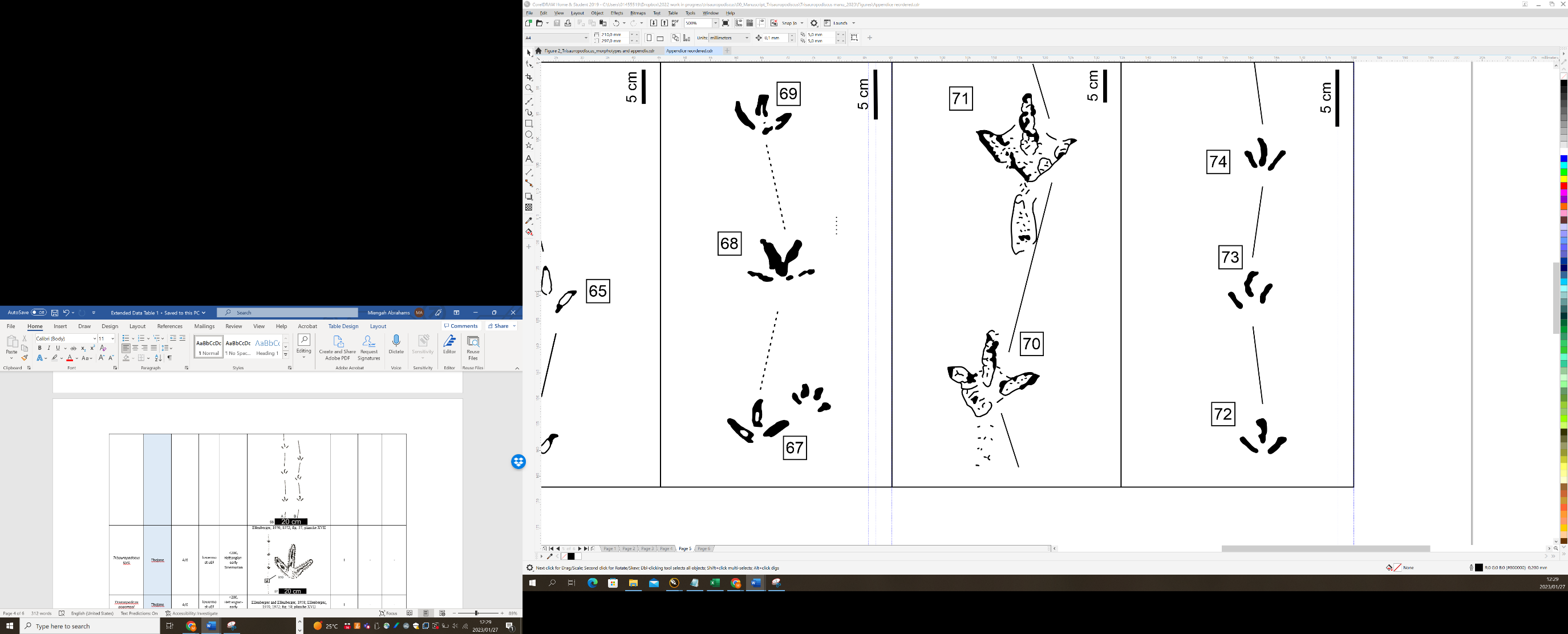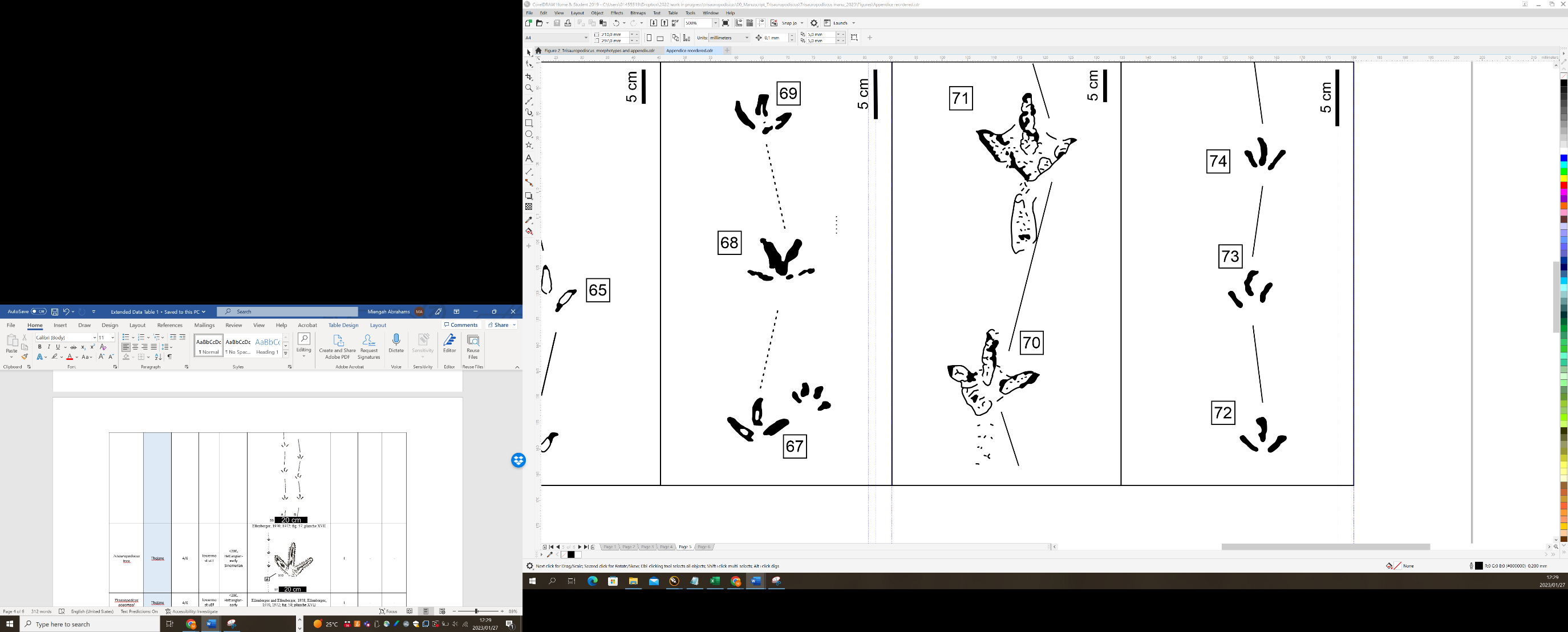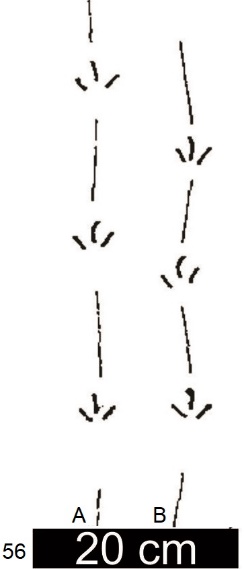 | II | - | - |
| *Trisauropodiscus*  *levis* | Thejane | A/6 | lowermost uEF | <200, Hettangian-early Sinemurian | Ellenberger, 1970; 1972; fig. 57; planche XVII  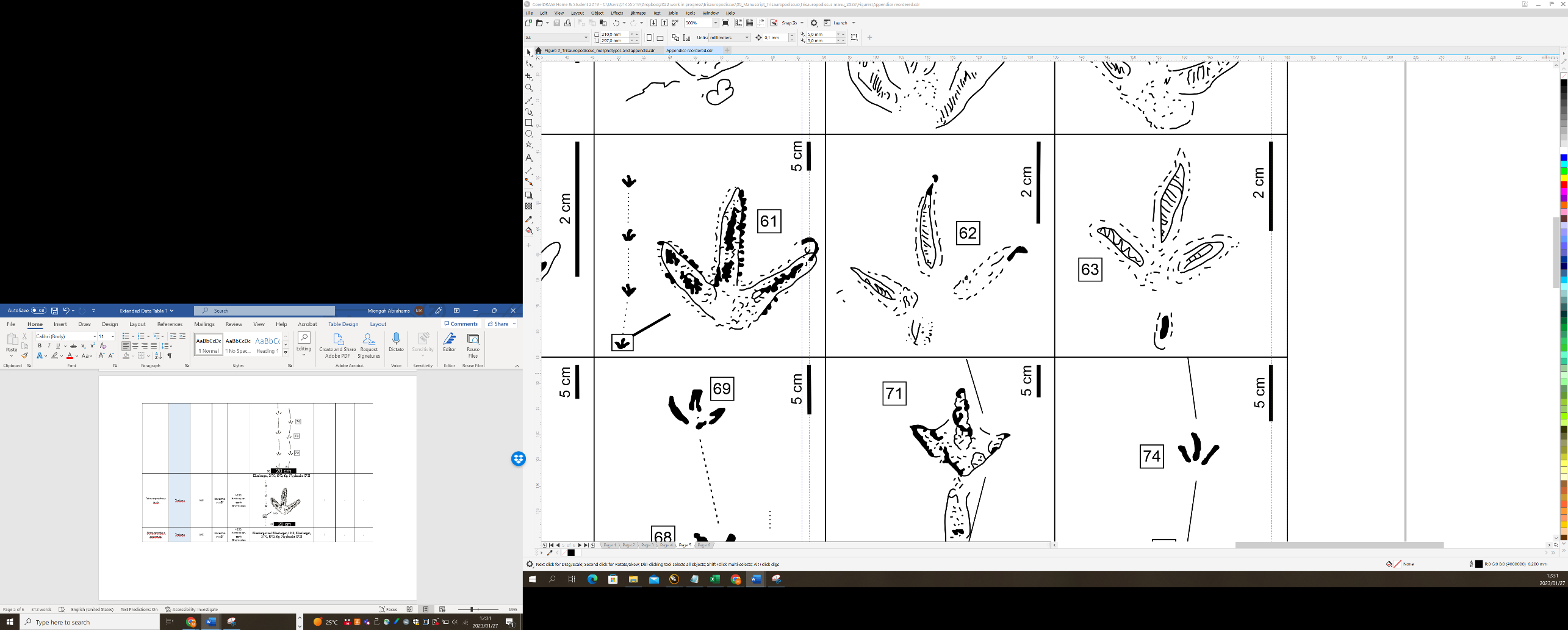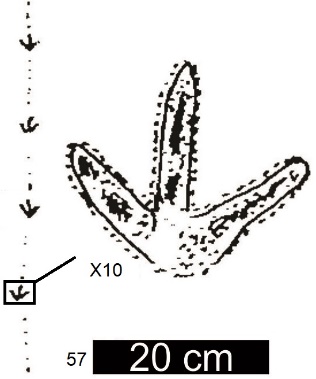 | I | - | - |
| *Tirsauopodicus*  *popompoi* | Thejane | A/6 | lowermost uEF | <200, Hettangian-early Sinemurian | Ellenberger and Ellenberger, 1958; Ellenberger, 1970, 1972; fig. 58; planche XVII  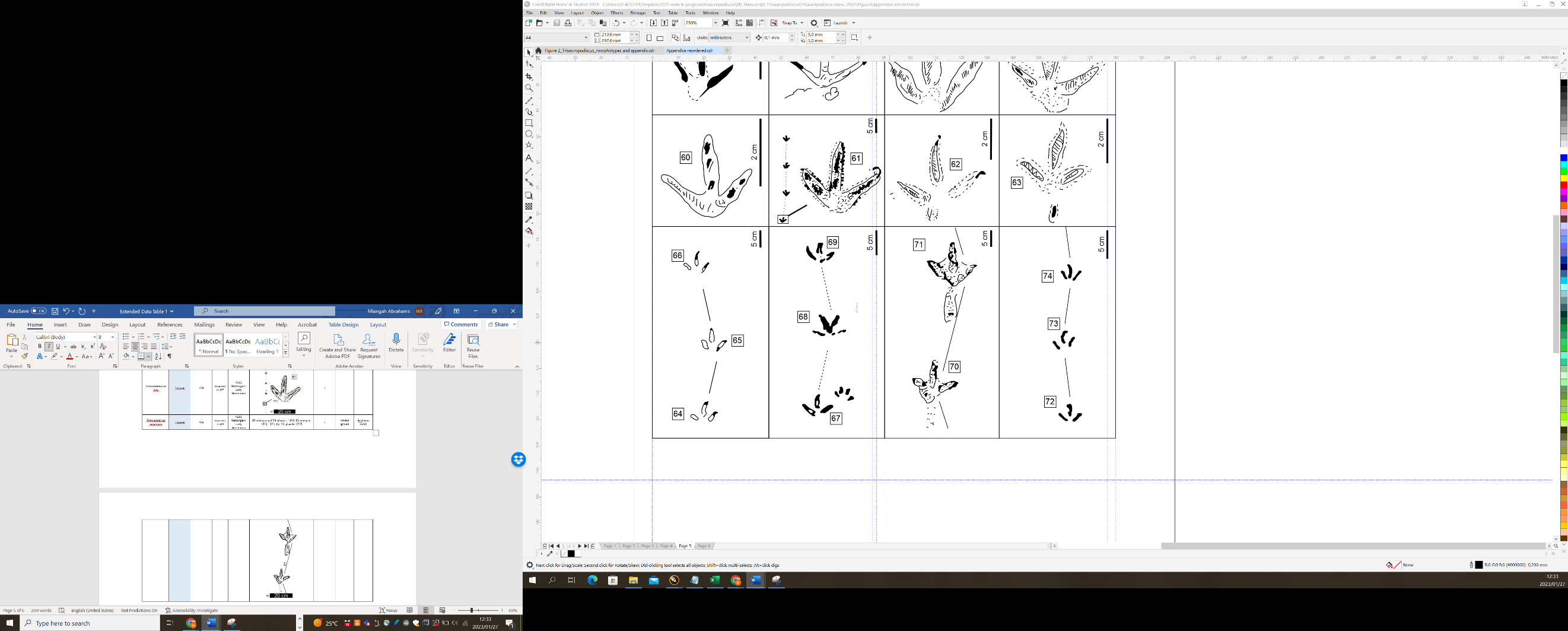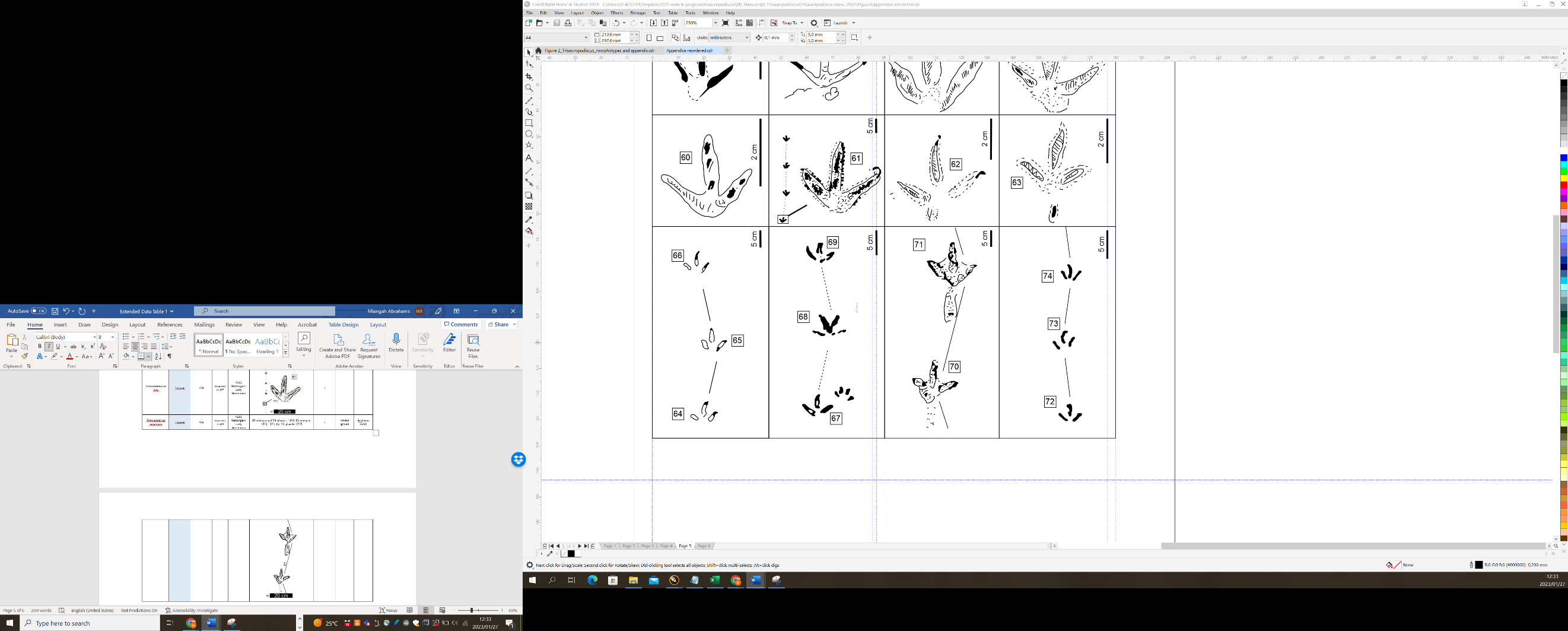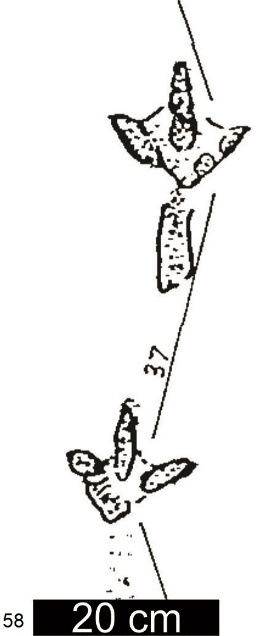 | I | LES068 @UoM | (lost from UoM) |
|  | Thejane | A/6 | lowermost uEF | <200, Hettangian-early Sinemurian | **Cast material**  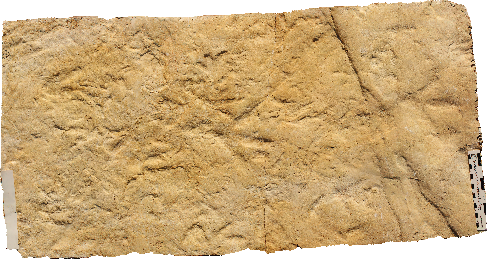  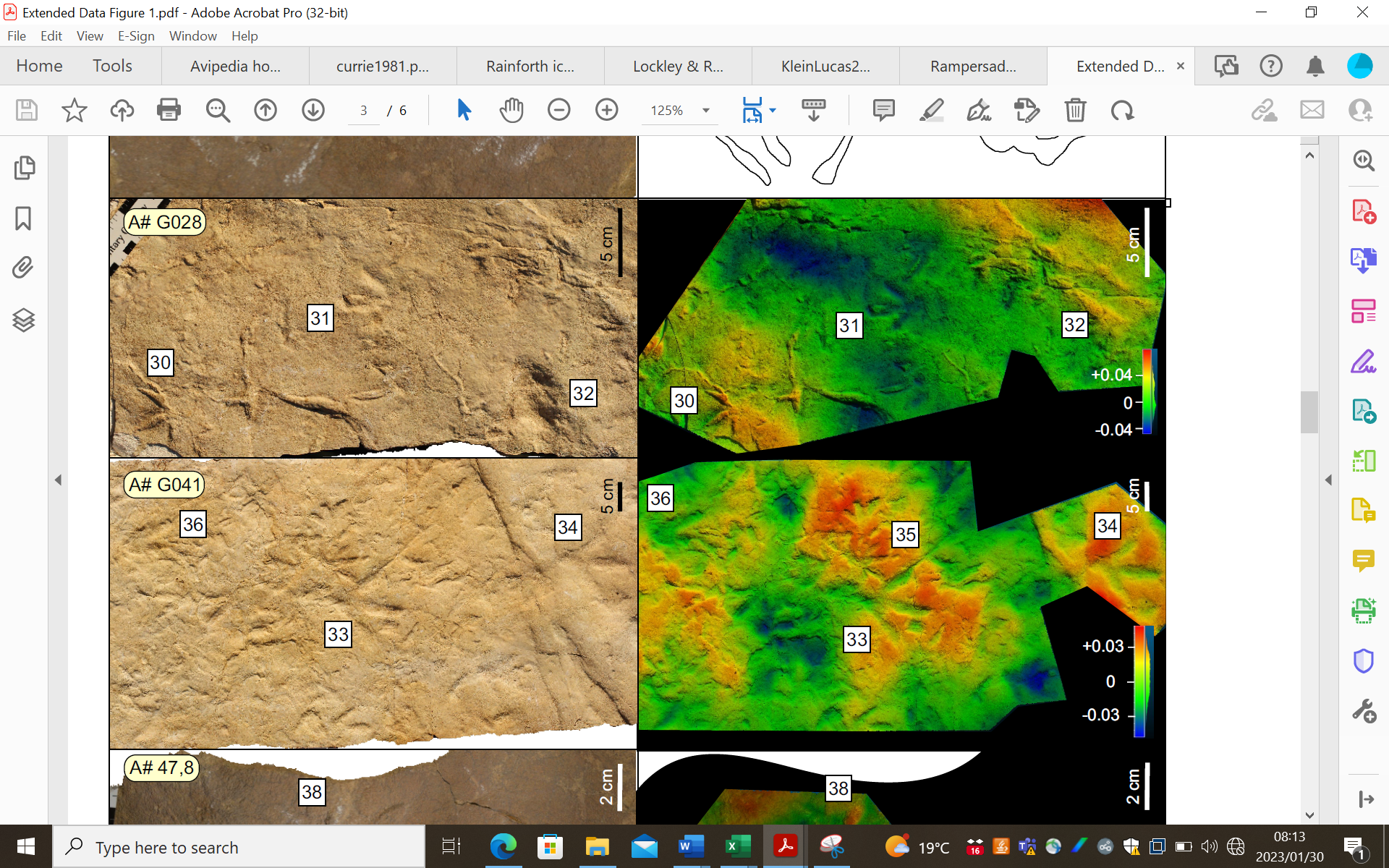  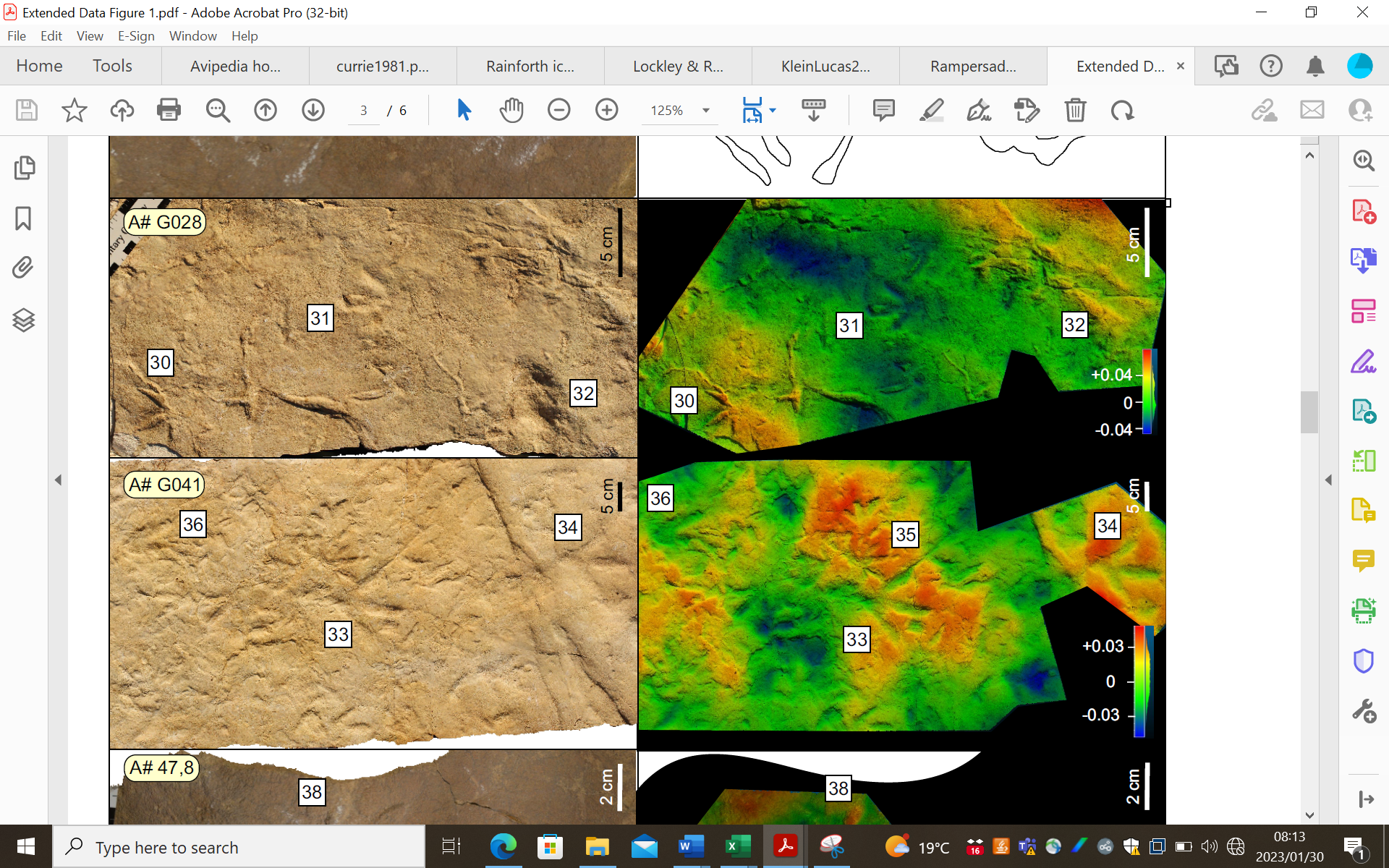  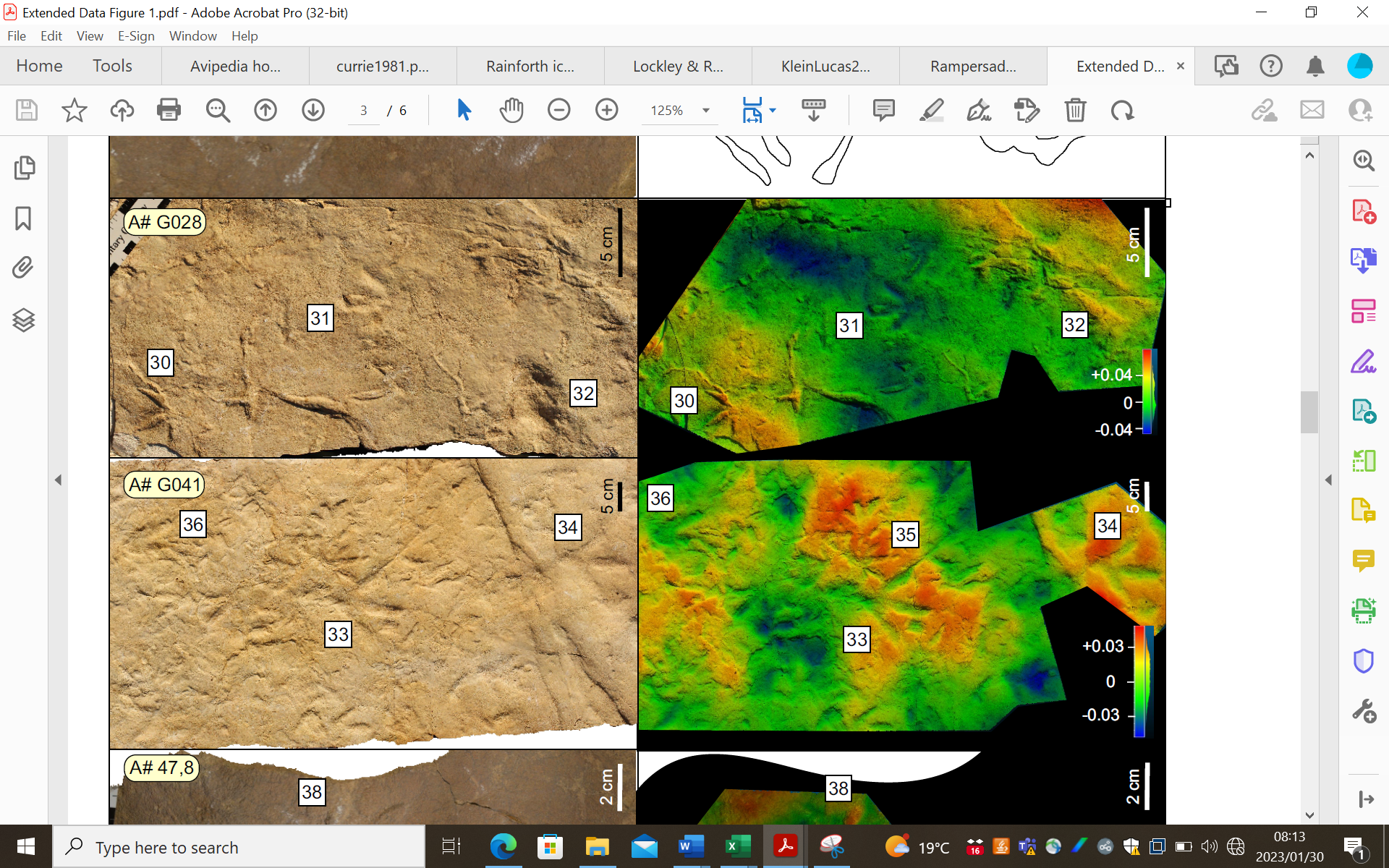  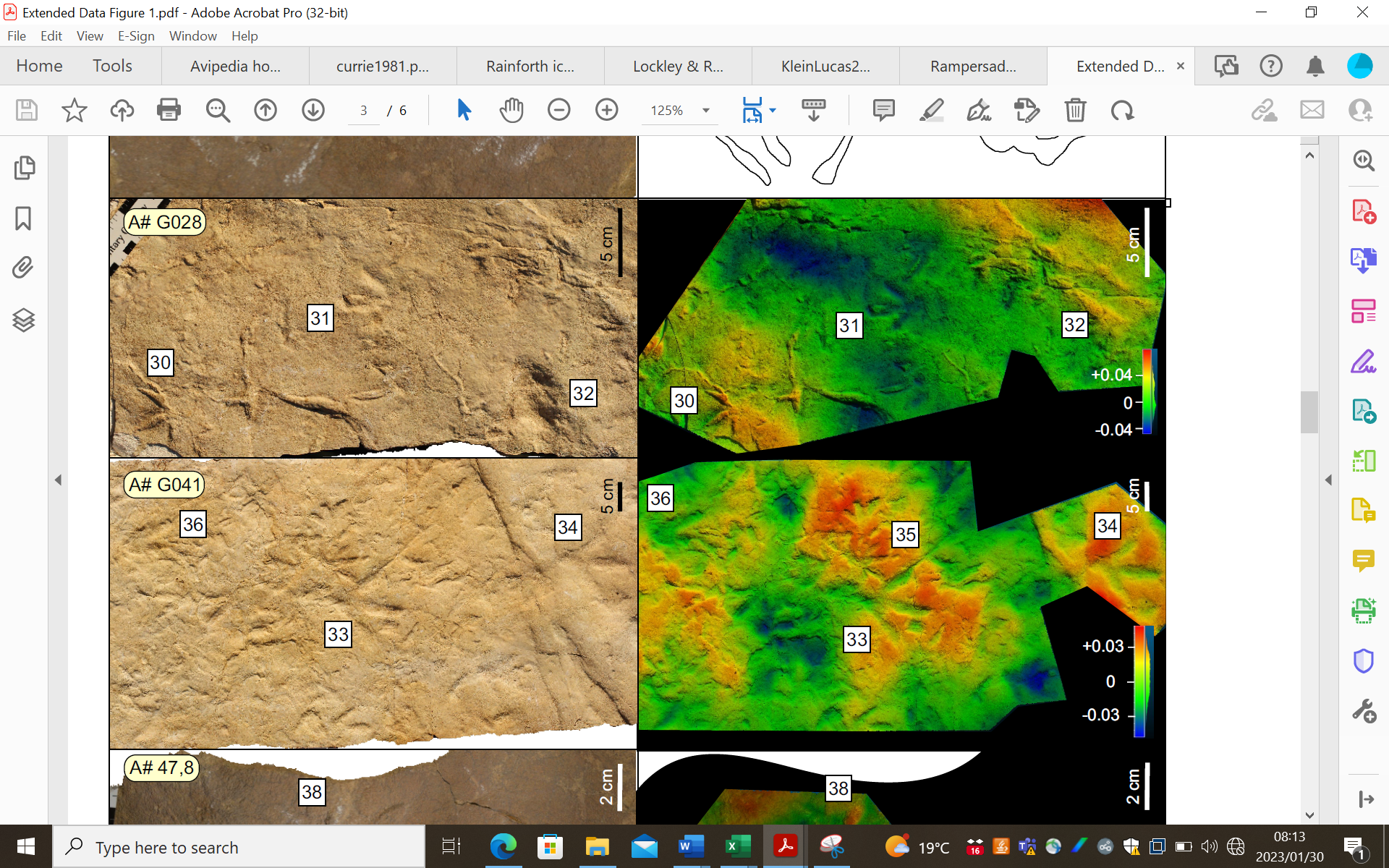 | I | BMG041 | MM&A |
|  | Thejane | A/6 | lowermost uEF | <200, Hettangian-early Sinemurian | 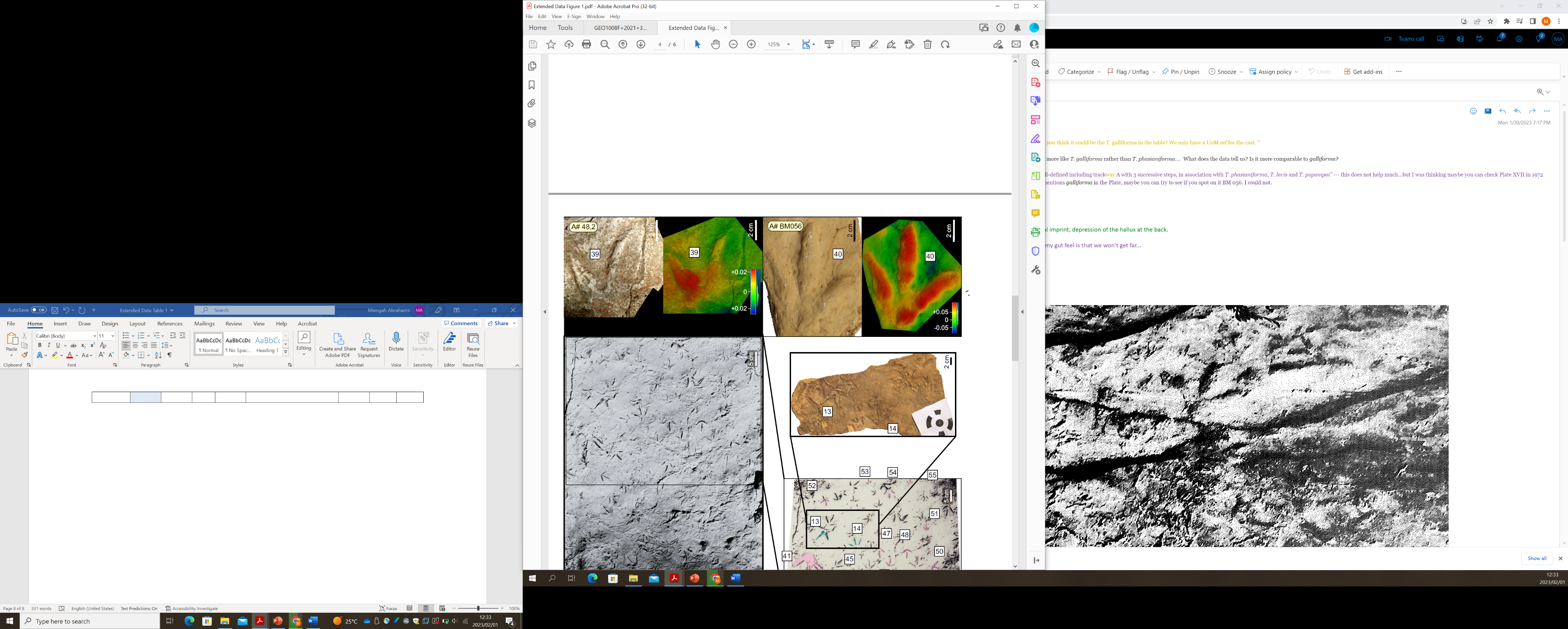 | I | BM056 | MM&A |
